# Morphological, phenological, and transcriptional analyses provide insight into the diverse flowering traits of a mutant of the relic woody plant *Liriodendron chinense*

**DOI:** 10.1038/s41438-021-00610-2

**Published:** 2021-08-01

**Authors:** Yu Sheng, Zhaodong Hao, Ye Peng, Siqin Liu, Lingfeng Hu, Yongbao Shen, Jisen Shi, Jinhui Chen

**Affiliations:** 1 Key Laboratory of Forest Genetics & Biotechnology of Ministry of Education, Co-Innovation Center for Sustainable Forestry in Southern China, Nanjing Forestry University, Nanjing, China; 2 College of Biology and the Environment, Nanjing Forestry University, Nanjing, China; 3 Southern Tree Seed Inspection Center National Forestry Administration, Nanjing Forestry University, Nanjing, China

**Keywords:** Flowering, Plant morphogenesis, Transcriptomics, Non-model organisms

## Abstract

Flowering is crucial to plant reproduction and controlled by multiple factors. However, the mechanisms underlying the regulation of flowering in perennial plants are still largely unknown. Here, we first report a *super long blooming 1* (*slb1*) mutant of the relict tree *Liriodendron chinense* possessing a prolonged blooming period of more than 5 months, in contrast to the 1 month blooming period in the wild type (WT). Phenotypic characterization showed that earlier maturation of lateral shoots was caused by accelerated axillary bud fate, leading to the phenotype of continuous flowering in *slb1* mutants. The transcriptional activity of genes related to hormone signaling (auxin, cytokinin, and strigolactone), nutrient availability, and oxidative stress relief further indicated active outgrowth of lateral buds in *slb1* mutants. Interestingly, we discovered a unique *FT* splicing variant with intron retention specific to *slb1* mutants, representing a potential causal mutation in the *slb1* mutants. Surprisingly, most *slb1* inbred offspring flowered precociously with shorter juvenility (~4 months) than that (usually 8–10 years) required in WT plants, indicating heritable variation underlying continuous flowering in *slb1* mutants. This study reports an example of a perennial tree mutant that flowers continuously, providing a rare resource for both breeding and genetic research.

## Introduction

Flowering, a phase change from vegetative to reproductive growth, is the most dramatic phase change and critical developmental switch in a plant’s life cycle. Flowering plants, as sessile organisms living in constantly changing environments, have evolved sophisticated mechanisms to sense changing environments and adjust development accordingly to ensure the optimal timing of flowering, thereby maximizing their reproductive success and ensuring the continuation of their species^1^.

Floral induction, floral meristem identity and flower morphogenesis have been described as the three major steps in the flowering process^2^. Decades of research in model plant systems have provided most of what we currently know about the regulatory mechanism of flowering timing. Multiple major genetic pathways have been proposed to integrate both external cues (photoperiod, cold, and ambient temperature) and endogenous cues (gibberellin, age, and carbohydrate state) to determine the timing of flower initiation^3–5^. In *Arabidopsis thaliana*, these flowering pathways form a complex, crosstalk, feedback loop regulatory network and converge to coordinately regulate several genes named “flower pathway integrators”^6,7^. In addition, recent advances have integrated epigenetic regulation factors (chromatin remodeling, histone modification, and miRNAs) into known flowering pathways, which synergistically contribute to the decision-making process for flowering^8–10^. The genetic control of floral organ number, arrangement and initiation timing has been well explained by the ABCDE model^11^, which was initially proposed based on extensive studies performed on floral homeotic mutants of the model plants *A. thaliana* and *Antirrhinum. majus*^12^. Five classes of homeotic genes (A, B, C, D, and E) function cooperatively to control the development of flower whorls or gametophytes^11,12^.

Perennial plants flower repeatedly in yearly cycles and are likely to be regulated via mechanisms that are distinct from those in annuals. Variants of economically important ornamentals and crops, such as some cultivars of *Dimocarpus longan*^13^, citrus^2^, rose and strawberry^14^, display the interesting character of flowering continuously in suitable seasons, which is extremely valuable for long-term fruit or flower production. Limited case studies have provided insights into this biological feature of continuous flowering. Mutation of *KSN* (ksncopia), a homolog of *A. thaliana TERMINAL FLOWER1* (*TFL1*), confers competency of recurrent blooming in continuously flowering rose and *Fragaria vesca*^14^. *PERPETUAL FLOWERING1* (*PEP1*), an ortholog of the *A. thaliana* gene *FLOWERING LOCUS C* (*FLC*), limits flowering duration by promoting the return to vegetative growth after flowering in *Arabis alpina*, and the *pep1* mutant shows a long flowering duration of more than 12 months^15^.

The genus *Liriodendron*, part of the magnolia family (Magnoliaceae), contains only one pair of sister species that are separated between East Asia (*L. chinense*) and eastern North America (*L. tulipifera*)^16^. With its straight trunk, conical crown, distinctive leaf shape, and tulip-shaped flowers, *Liriodendron* is an excellent ornamental tree for landscaping^17^. In addition, *Liriodendron* has been widely planted as an industrial timber species due to its rapid growth and versatile wood with excellent working properties^17,18^. It is also valued as a honey tree, as a source of food for wildlife and for its potential medicinal properties^17,19^. Contrary to *L. tulipifera*, which is widely distributed in eastern North America, *L. chinense* is an endangered plant listed in the China Plant Red Data Book^20^. Therefore, acceleration of the breeding program for this endangered woody plant, based on further in-depth understanding of the molecular mechanisms controlling its development, especially those of its reproductive traits, is of great importance.

Here, we report for the first time a spontaneous mutant of *L. chinense*, *super long blooming1* (*slb1*), and combine morphological identification, phenological observation, and transcriptional profiling to comprehensively characterize its developmental features and explore the transcriptional regulatory mechanisms possibly underlying its unique phenotype.

## Results

### Flowering phenology shifts in the continuously flowering *slb1* mutants of *L. chinense*

**Fig. 1:**
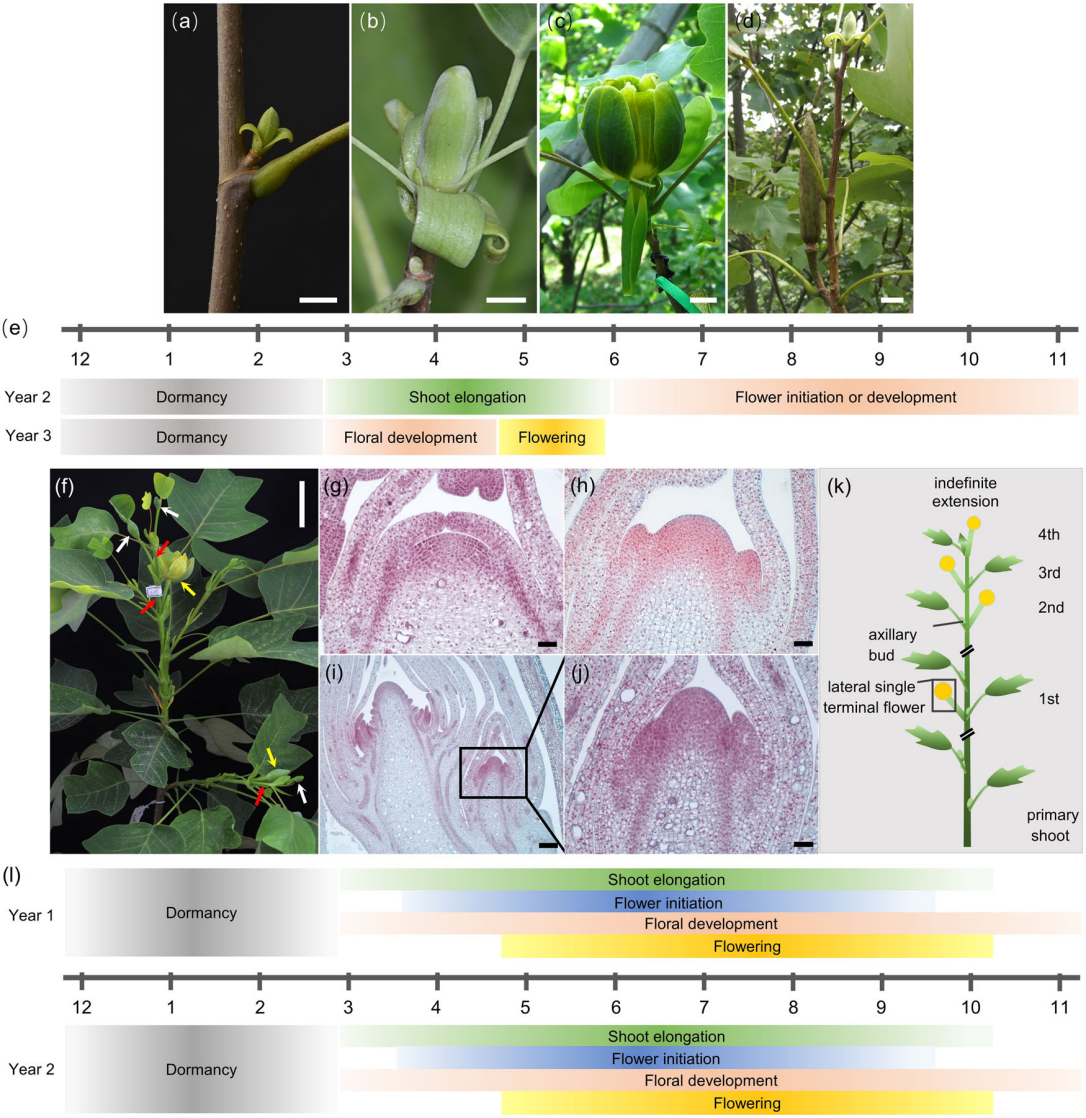
Comparison of blooming modes in *slb1* mutants and WT plants. **a** Vegetative bud growing from a leaf axil. **b** Mixed bud situated on the short shoot apex. **c** Single terminal flower. **d** Aggregate fruit in the shoot apex and a one-year lateral branch (expected to flower the next year) arising from an axillary bud sitting below the mixed bud on a shoot tip. **e** Diagram showing the developmental progress of a flower branch in WT *Liriodendron* (axis indicating months 1–12 of the year) (according to Fan et al., 1992^19^; You & Fan, 1993^20^), illustrating the flower transition in June to August and flowering in late April to mid-July of the next year. **f** Flower branch of *slb1* plant, showing additional branches (red arrow) developing from axillary buds below the older terminal flowers (yellow arrow), followed by the generation of new lateral single terminal flowers (white arrow). **g**–**j** Histological observation of *slb1* floral meristem differentiation. **g** Vegetative bud. **h** Flower initiation. **i** Terminal flower with lateral floral structure (boxed). **j** Partially enlarged detail of (**i**). **k** Schematics showing the observed indefinitely extending flower branch in *slb1*. Note that the number of internodes between the two most proximal flowers arising on the sympodial shoot is not unified before every vegetative node changes into a flower. **l** Diagram showing the observed growing habits of *slb1* (axis indicating months 1–12 of the year). Characteristics include continuous shoot elongation, flower initiation, organogenesis and maturation for as long as the reproductive period of one year lasts. Scale bars: (**a**) and (**b**) 0.5 cm, (**c**) and (**d**) 1 cm; (**f**) 5 cm; (**g**) and (**h**) 100 µm; (**i**) 200 µm; (**j**) 50 µm

To understand the botanical features of *slb1* mutants for further genetic study of flowering behavior, we compared the growth habits of *slb1* mutants and wild-type (WT) plants. *Liriodendron* possesses two bud types, a vegetative bud located in the axil of the leaf (Fig. 1a) and a mixed bud located in the shoot apex (Fig. 1b), both of which are covered with scales. Mixed buds may differentiate from vegetative buds and can in turn give rise to axillary buds. Vegetative buds residing on the upper nodes are formed in the first year and then undergo activation and elongation, forming new shoots in the next spring (Fig. 1e). Then, the mixed apical buds on these new shoots will undergo a transition from the vegetative apex to the floral apex in early June (Fig. 1e), accompanied by the formation of new axillary vegetative buds. By the third year, flower buds differentiated during the last year will bloom from April to May (Fig. 1c, e). Overall, it normally takes three years from the initiation of a mixed bud to its full blooming^21,22^ (Fig. 1e).

The continuously flowering *slb1* mutants have the same first flowering date in April as WT plants (Fig. 1e, l), during which flowers differentiate from flower buds that formed in the previous growing season. Then, WT plants will normally not flower again, except for a rarely observed second flowering event in October of the same year^21^. In contrast, *slb1* mutants exhibit a greatly prolonged flowering duration of almost six months from mid-April to early October (Fig. 1l). More importantly, while WT plants undergo the reproductive transition from June to August in the second year after the formation of vegetative buds, i.e., the precursor of flower buds (Fig. 1e), flower initiation of *slb1* mutants occurs from the first growing season (early March) until late Autumn (late September) (Fig. 1l). In *slb1* mutants, both flower initiation and blooming can occur within the same season (Fig. 1f, l). Continued flower initiation was further confirmed by histological observations of weekly bud sampling (Fig. 1i, j). Developmental events of tepal primordium initiation or tepal formation were observed in early March, April, June, July, and late September in *slb1* mutants (Fig. 1h, j). A prominent characteristic of *slb1* mutants is the intensive and extensive overlap between vegetative and reproductive development (Fig. 1f, k, l). Continuous floral transition in *slb1* mutants occurs during most of the year, as long as environmental conditions (e.g., temperature and light) are sufficient to allow reproduction.

### The developmental nonrobustness of *slb1* flowers

**Fig. 2:**
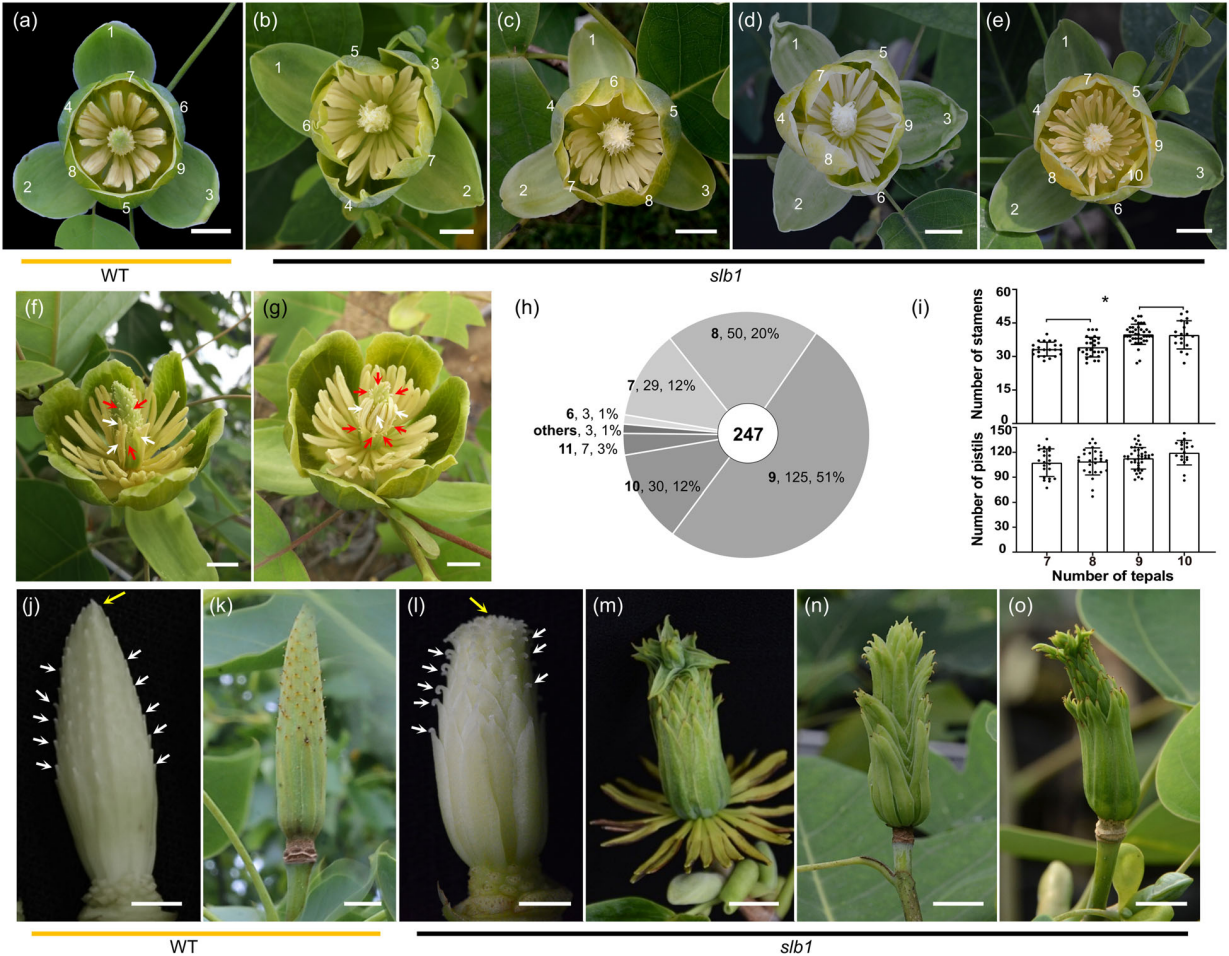
*slb1* mutants show dramatic variability in floral architecture. **a** WT flower, with a total of 9 tepals in 3 whorls. **b**–**e** *slb1* flowers with various tepal number phenotypes. **b** A 7-tepal flower with loss-of-radial symmetry: only 2 tepals arise oppositely in the outermost whorl. **c** An 8-tepal flower missing one tepal in the second whorl. **d** A 9-tepal flower identical to the WT in flower architecture. **e** A 10-tepal flower with an extra fourth whorl with only one tepal. **f**–**g** *slb1* flowers showing loss of organ boundaries between stamens (white arrow) and carpels (red arrow). **f** Inner stamens fused with nearby carpels at their base. **g** Ectopic stamens occupying the expected position of carpels. **h** Pie chart showing the frequency of *slb1* flowers with a certain tepal number (ranging from 6 to 11, black bold digit) from a total of 247 flowers. A high proportion (~49%) of *slb1* flowers have an abnormal tepal number, with ~51% having the normal case of nine tepals. **i** Histograms showing the petal number on the *x*-axis and the corresponding stamen and pistil numbers on the y-axis. Significance levels are marked by asterisks: *P < 0.05. **j**–**o** Morphological differences between WT and *slb1* gynoecia (**j** and **l**, respectively) and fruits (**k** and **m**–**o**, respectively). Arrows indicate the gynoecium apex (yellow) and pistil apex (white). Note the incomplete aggregate status of fruits of *slb1* gynoecia (**l**) and fruits (**m**–**o**). Scale bars: (**a**–**g**), (**k**), (**m**–**o**) 1 cm; (**j**) and (**l**) 0.5 cm

To gain further insights into the variations in ontogenetic development, we detected the structure of flowers in *slb1* mutants and WT plants. WT *Liriodendron* has bisexual flowers with a cyclic group of tepals that constitute three strictly alternating trimerous whorls and an androecium of many stamens surrounding a gynoecium of multiple carpels on a common receptacle (Fig. 2a). In *slb1* mutants, we observed high diversity in patterns of floral construction, including variations in tepal number per whorl, flower symmetry, organ fusion, and boundary formation.

The tepal number of *slb1* mutants varies from seven to ten, with a few outliers having six or eleven (Fig. 2b–e, h and Supplementary Fig. 1). A high proportion (up to 49%) of the 247 examined flowers displayed tepal number variation (Fig. 2h), and importantly, multiple types of tepal number variations could be found within an individual *slb1* plant, even on a single flower shoot. Specifically, *slb1* flowers with seven tepals showed an irregular arrangement of tepals, with all three whorls having lost their typical radial symmetry, only two tepals within the outermost flower whorl and blurred boundaries between the inner two whorls (Fig. 2b). Eight-tepal *slb1* flowers lack one tepal within the intermediate whorl (Fig. 2c), while an extra, much smaller, tepal develops on the inner side of the innermost tepal whorl in ten-tepal flowers (Fig. 2e). However, three alternating trimerous whorls are still clearly recognizable despite the deletion or insertion of one tepal in eight- or ten-tepal *slb1* flowers (Fig. 2c, e).

*Liriodendron* flowers contain another two whorls inside the cyclic tepal group, i.e., the innermost whorl, including a gynoecium that consists of multiple pistils, and the second surrounding whorl, including an androecium that consists of multiple stamens. The numbers of pistils and stamens also varied across individual *slb1* flowers (Fig. 2i). Furthermore, we observed positive correlations between tepal number and pistil/stamen number (Fig. 2i and Supplementary Table 1). These results indicate that an abnormally formed floral meristem, instead of a homeotic transition between different floral organs, leads to the variability of *slb1* floral architecture.

In WT plants, organ transformation between the androecium and gynoecium is rarely observed because these zones have distinct and stable organ boundaries and stamens and pistils are morphologically different. However, several *slb1* flowers showed occasional blurring of organ boundaries between stamens and carpels. We noted that a small number of inner stamens (Fig. 2f, white arrow) would fuse with nearby carpels (Fig. 2f, red arrow) at their base or that stamens (Fig. 2g, white arrow) would ectopically occupy the expected position of carpels (Fig. 2g, red arrow).

WT *L. chinense* flowers always have a long, conical gynoecium with carpels that are free at the apex and adnate to nearby carpels at the base (Fig. 2j). Meanwhile, the aggregate fruit, as a subsequent mature organ derived from the gynoecium, has a compact and long, nearly conical structure (Fig. 2k). In contrast, in *slb1* mutants, we found that the carpel apex bends downwards (Fig. 2l, white arrow) and that carpels are disorderly arranged on the gynoecium tip (Fig. 2l, yellow arrow), resulting in a loosened aggregate fruit structure (Fig. 2m–o). Overall, these results show that *slb1* displays extensive floral organ variations, some of which even lead to abnormal fruit development, suggesting the developmental nonrobustness of floral meristems in *slb1* mutants.

### Global transcriptome analyses within and between genotypes

**Fig. 3:**
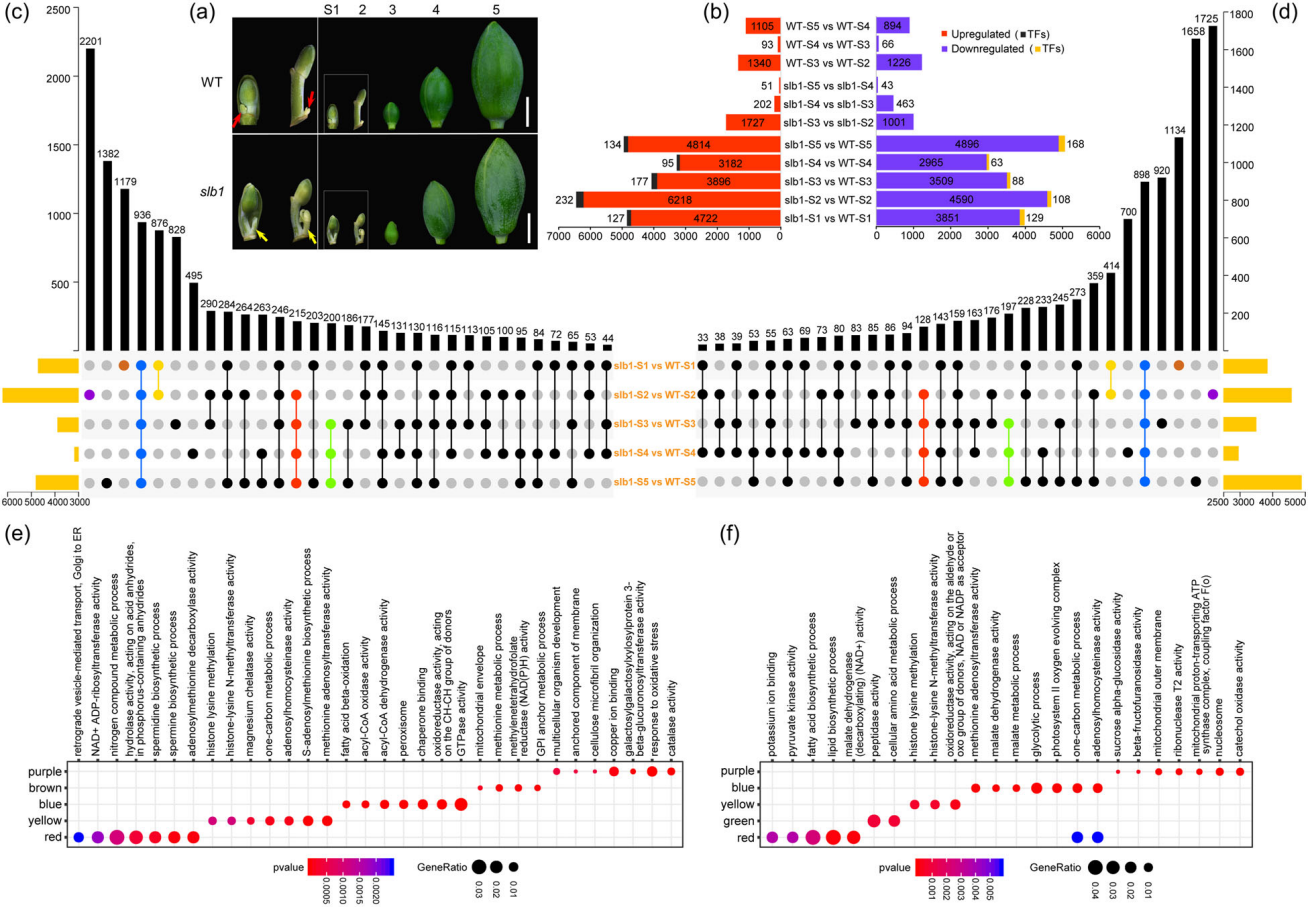
Plant samples for RNA sequencing and total number of DEGs (fold change ≥ 2 or ≤ -2, Q-value < 0.05, FPKM ≥ 1) in pairwise comparisons of developmental stages between or within *slb1* and WT genotypes. **a** Plant materials for RNA-seq. S1: Autumn bud; S2: Spring bud. There was a slightly elongated lateral bud beneath the apical flower bud in S1 and S2 mixed buds in *slb1* (yellow arrow) compared to the WT (red arrow); S3-S5: flower buds at different developmental phases (~ 0.7, 2, and 3 cm in length, respectively). **b** Histogram illustrating the numbers of upregulated (red bars) or downregulated (blue bars) DEGs in pairwise comparisons as well as transcription factors (TFs) up/downregulated (black and brown bars, respectively) between and within genotypes at each developmental stage. **c** UpSet plot of common and unique upregulated DEGs in five time-point pairwise comparisons between *slb1* and WT. **d** UpSet plot of specific and shared downregulated DEGs in different developmental stages between genotypes. **e** GO enrichment of unique or common upregulated DEGs labeled colorfully in (**c**). **f** GO enrichment of unique or common downregulated DEGs marked colorfully in (**d**). Bars: 1 cm

To identify the transcriptional regulatory mechanisms underlying the developmental defects in *slb1* mutants of *L. chinense*, we extracted total RNA from mixed buds in autumn and spring and flower buds at three different developmental stages of *slb1* mutants and WT plants (Fig. 3a) for library construction and performed a comparative time-course transcriptional profiling analysis using PacBio Iso-Seq and Illumina RNA-seq. Two sets of full-length transcriptomes (G1 and F1), totaling 6,981,229 (5,124,340 and 1,856,889, respectively) subreads, were generated (Supplementary Table 2). After merging and error correction, 836,928 circular consensus sequences (CCSs) were obtained, and 49% (355,451) of the CCSs were classified as full-length nonchimeric reads (FLNCs) (Supplementary Table 2). The FLNCs were then subjected to clustering and further hybrid correction using PacBio and Illumina reads and redundancy removal. Finally, 146,826 unigenes with lengths ranging from 270 to 11,816 bp (average ~2,572 bp) were obtained (Supplementary Table 3).

Pairwise comparisons between adjacent stages within the same genotype or between genotypes at the same developmental stage were performed to identify differentially expressed genes (DEGs), using the following thresholds: fold-change ≥ 2 or ≤ -2, Q-value < 0.05, and FPKM cutoff ≥1. The results showed a much higher number of DEGs between genotypes than between successive developmental stages (Fig. 3b), suggesting a great deal of developmental heterogeneity in flower development between *slb1* mutants and WT plants. We found that multiple transcription factor (TF) families, including FAR1, bHLH, MIKC_MADS, MYB, bZIP, HD-ZIP, ARF, C2H2, C3H, and SBP, were differentially expressed in *slb1* mutants compared to WT plants at all developmental stages (Supplementary Fig. 2), indicating that developmental heterogeneity is at least partially caused by differential transcriptional regulation mediated by distinct combinations of TFs.

To further analyze the functionality of DEGs identified in all five pairwise comparisons, functional enrichment analyses were performed. Genes downregulated in *slb1* mutants compared with WT plants were significantly enriched in GO terms related to photosynthesis and fatty acid biosynthesis (Supplementary Fig. 3). Furthermore, both down- and upregulated DEGs across most of the five pairwise comparisons were found to be distinctly enriched for GO terms related to GTPase activity (Supplementary Fig. 3). A similar result was also observed for the functional categories of unique and common DEGs from pairwise comparisons between the two genotypes (Fig. 3c, d). Specifically, 936 commonly upregulated DEGs were overrepresented for a GO term related to GTPase activity and fatty acid metabolism, i.e., “fatty acid beta-oxidation” and “acyl-CoA oxidase/dehydrogenase activity” (Fig. 3e), and KEGG pathways of “biosynthesis of unsaturated fatty acid” and “fatty acid degradation” (Supplementary Fig. 4a). Meanwhile, 898 common downregulated DEGs were enriched in GO terms of “glycolytic process” and “photosystem II oxygen evolving complex” (Fig. 3f). Overall, functional classification terms involving substance and energy metabolic processes were significantly enriched for both up and downregulated DEGs, implying extremely large variations in carbohydrate production and various energy-consuming mechanistic activities between *slb1* mutants and WT plants.

### Transcriptional comparisons across floral initiation and flower development between *slb1* mutants and WT plants

**Fig. 4:**
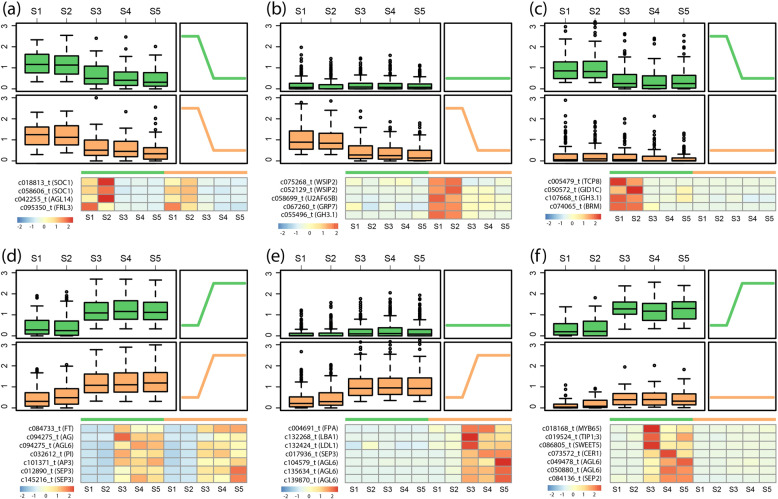
Developmental comparisons between *slb1* mutants and WT plants. All five stages were classified into two distinct developmental phases, i.e., floral initiation (Phase I, including the S1 and S2 stages) and floral development (Phase II, including the S1, S2, and S3 stages). Phase-specific genes were identified by correlation with the perfect modules, Phase I-specific expressed genes in *slb1* mutants (**b**, 174 transcripts), WT plants (c, 109 transcripts), or both of them (**a**, 68 transcripts) and Phase II-specific expressed genes in *slb1* mutants (**e**, 288 transcripts), WT plants (**f**, 57 transcripts), or both of them (**d**, 231 transcripts). A box plot of the gene expression levels (log_10_(RPKM + 1)) for each module is shown with a heatmap of selected gene expression below

To further explore the transcriptional mechanisms underlying continuous flowering in *slb1* mutants, we divided all samples into two developmental phases: floral initiation, including the S1 and S2 stages (Phase I), and floral development, including the S3, S4, and S5 stages (Phase II). For genes that were specifically highly expressed in Phase I in both *slb1* mutants and WT plants, we identified two transcripts of *SUPPRESSOR OF OVEREXPRESSION OF CO 1* (*SOC1*) (Fig. 4a), an integrator of multiple flowering pathways, including the autonomous, gibberellin (GA), vernalization, photoperiod, and age-related pathways^23,24^. Interestingly, for genes that were specifically highly expressed in Phase I only in *slb1* plants, we identified two genes, i.e., *U2 SMALL NUCLEAR RIBONUCLEOPROTEIN AUXILIARY FACTOR 6B* (*U2AF65B*) and *GLYCINE RICH PROTEIN 7* (*GRP7*) (Fig. 4b), that regulated flowering time via the floral repressor *FLC*^25,26^.

In addition, *FLOWERING LOCUS T* (*FT*), the florigen gene, was observed to be specifically highly expressed in Phase II in both *slb1* mutants and WT plants (Fig. 4d). Meanwhile, we found that several *AGAMOUS-LIKE 6* (*AGL6*) transcripts, which positively and negatively regulate *FT* and the floral repressor *FLC*, respectively^27^, were either specifically highly expressed in Phase II in *slb1* mutants (Fig. 4e), WT plants (Fig. 4f), or both of them (Fig. 4d). In addition, we found that several ABCs of floral homeotic genes, such as *AGAMOUS* (*AG*), *SEPALLATA3* (*SEP3*), showed a similar expression pattern to *AGL6* (Fig. 4d, e), consistent with the transcriptional pattern observed during flower development.

Collectively, two major flowering promoter genes, *SOC1* (two transcripts) and *FT* (one transcript), were specifically highly expressed in Phases I and II, respectively, with similar expression patterns between *slb1* mutants and WT plants (Fig. 4a, d). However, the floral repressor *FLC*, a MADS-box transcriptional regulator, was a potential target since its regulators, *U2AF65B* and *GPR7*, were highly expressed in Phase I only in the *slb1* mutant (Fig. 4b). To provide comprehensive insights into the MADS-box gene family, we identified and profiled the expression of all MADS-box genes in *slb1* mutants and WT plants. For ABCs of floral homeotic genes, such as *AG*, *SEP*, and *AP3*/*PI* clade, *slb1* mutants and WT plants both possessed transcripts with specifically high expression in Phase II (Supplementary Fig. 5). Nevertheless, we did not detect any transcripts of *FLC* in this transcriptome dataset (Supplementary Fig. 5) or in the *Liriodendron* genome^16^, consistent with the findings of previous studies^28^.

### A unique *FT* transcript specific to *slb1* mutants

**Fig. 5:**
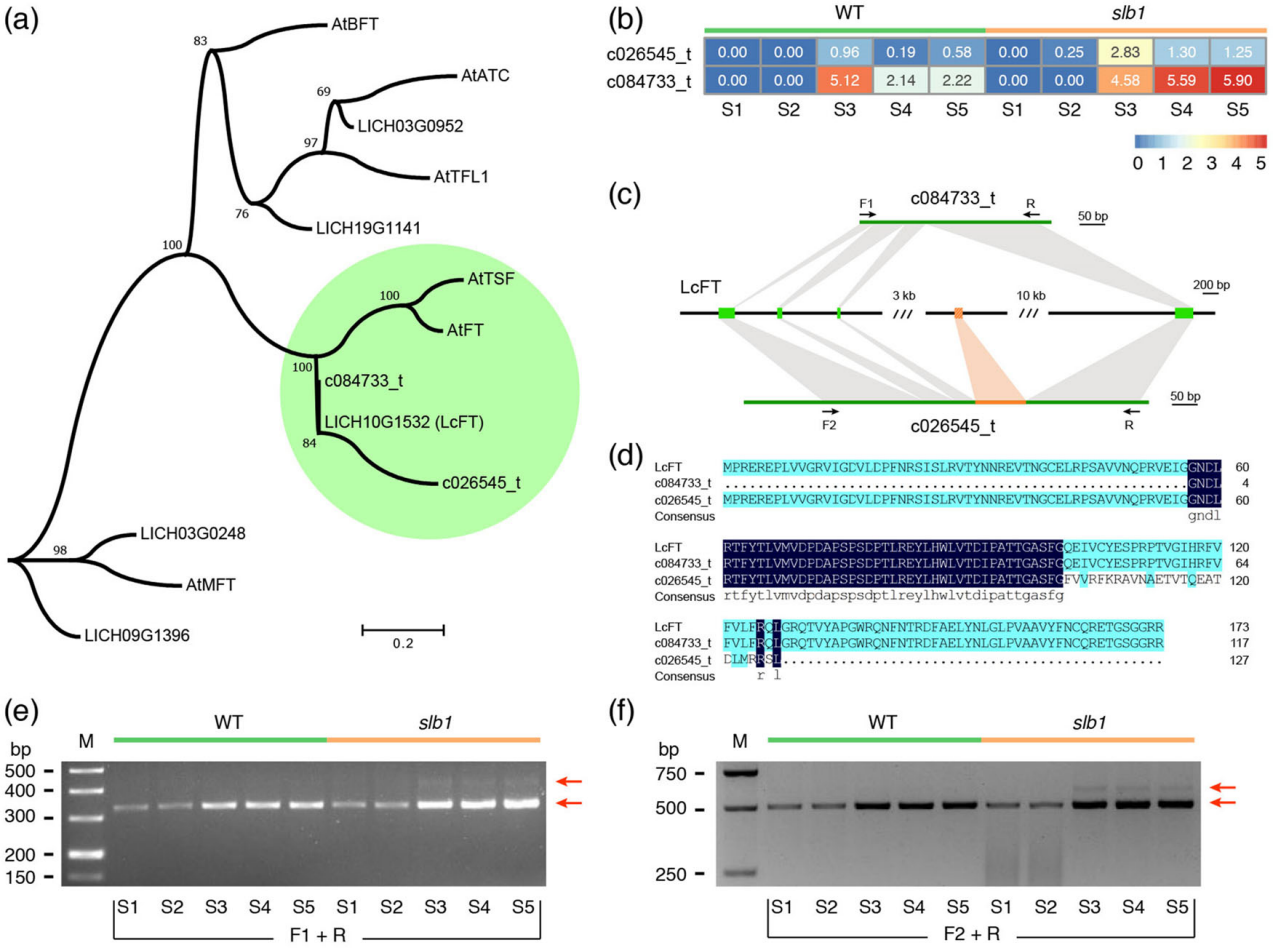
Identification of a unique *FT* splicing variant specific to *slb1* mutants. **a** Phylogenetic tree of the PEBP family obtained using the ML method. **b** Gene expression of two *FT* transcripts in *slb1* mutants and WT plants. **c** Schematic genomic structure of *FT* splicing variants. Green boxes, exons; dark line, untranslated regions; dark green lines, transcripts. **d** Protein sequence alignments of FT annotated in the *Liriodendron* genome and two transcripts detected in this transcriptome dataset. **e**, **f** The splicing variants detected by RT-PCR using two pairs of primers. F1 and F2 were specific forward primers for c084733_t and c026545_t amplification, respectively, and R was the reverse primer. M, marker; RT-PCR, reverse transcription-polymerase chain reaction; bp, base pairs

For *SOC1*, a total of two transcripts were identified with a Phase I-specific expression pattern as described above (Fig. 4d and Supplementary Fig. 5). Then, we asked whether the other flowering-promoting gene *FT* possessed additional transcripts. To this end, we identified the phosphatidylethanolamine-binding protein (PEBP) family, which contains six members in *Arabidopsis*, in this dataset and the *Liriodendron* genome (Fig. 5a). Only two transcripts of the PEBP family were detected in this transcriptome dataset, both of which encoded the FT protein, with one (c084733_t) mentioned above and the other (c026545_t) being specifically expressed in Phase II only in *slb1* mutants (Fig. 5b). Surprisingly, by aligning these two transcripts with the *Liriodendron* genome sequence, we found that the latter possessed an additional exon (Fig. 5c), leading to a diverged C-terminal region and truncating the c026545_t protein at a premature stop codon (Fig. 5d).

Detailed sequence analysis showed that the additional exon of c026545_t was matched to the intron region between the 3rd and 4th exons (Fig. 5c), possibly resulting from intron retention. In addition, the other transcript, c084733_t, seemed to have lost the N-terminal region (Fig. 5c), which was likely caused by sequencing and/or assembly errors. Thus, we designed two pairs of primers, as shown in Fig. 5c, to test whether the 5′-end of c084733_t was lost and whether intron retention in c026545_t truly existed. As expected, we detected the presence of the normal *FT* transcript using both primer pairs (Fig. 5e, f), indicating that the absence of the 5′-end in c084733_t was caused by assembly errors. Surprisingly, we detected a second variant, which was consistent in size with c026545_t with an additional 95 bp of nucleotides, in Phase II only in *slb1* mutants with a much lower transcript abundance compared to that for the normal *FT* transcript (Fig. 5e, f). In addition, we also detected the presence of the normal *FT* transcript in Phase I in both *slb1* mutants and WT plants (Fig. 5e, f), even though the transcript abundance was lower than that in Phase II.

### Detection of coexpression patterns of genes across different developmental phases

**Fig. 6:**
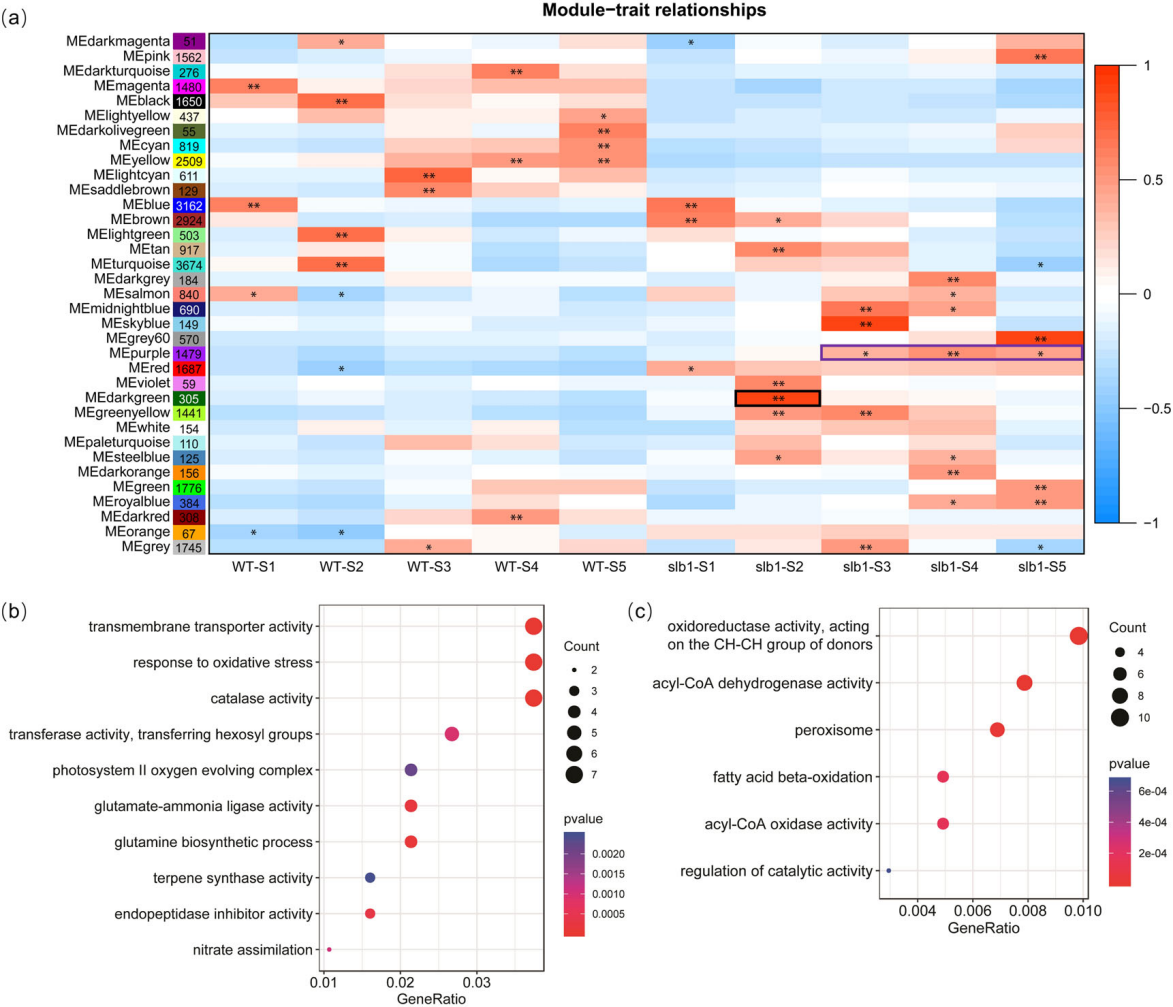
WGCNA of *slb1* and WT. Each row corresponds to a module labeled with a color, as in (**a**). Modules are distinguished by different colors that were arbitrarily assigned by the WGCNA package. Each column corresponds to a tissue type as indicated. The color of each cell at the row–column intersection indicates the correlation coefficient (R) between the module and the tissue type. *Significance at P < 0.05; **Significance at *P* < 0.01. **b** GO enrichment of the dark green module (black rectangle specific to the *slb1* S2 stage. **c** GO enrichment of the purple module (purple rectangle) specific to stages S3-S5

To identify specific genes that were highly associated with certain developmental stages, we obtained coexpressed gene sets via weighted gene coexpression network analysis (WGCNA). A total of 35 modules, labeled by different colors, were identified (Fig. 6a and Supplementary Fig. 6). We observed nine modules that positively correlated with *slb1* tissue obtained from flower stages S4 and/or S5 (Fig. 6a). KEGG enrichment analysis revealed that pathways related to the biosynthesis of diverse secondary metabolites, including terpenoid backbone, flavonoids, flavones, flavonols, and carotenoids, were enriched in the dark gray, midnight blue, gray 60, and dark orange modules (black-underlined in Supplementary Fig. 9). They linked metabolic activity to late flower developmental stages, i.e., flower maturation progression, such as flower color and/or volatiles production. The purple module, containing 1,479 genes, was specifically positively correlated with the S3-S5 stages of *slb1* flower development and was enriched in GO terms related to cellular redox activity, including “oxidoreductase activity”, “acyl-CoA oxidase/dehydrogenase”, “peroxisome”, and “fatty acid beta-oxidation” (Fig. 6a, c).

In addition, 305 genes in the dark green module were specifically positively correlated with the S2 stage of *slb1* mutants (Fig. 6a, labeled by a black box). GO enrichment analysis showed that transmembrane transporter-related genes were significantly enriched for this module (Fig. 6b), including a carbohydrate transporter (*POLYOL/MONOSACCHARIDE TRANSPORTER5*, *PLT5*) and an external inorganic phosphate transporter (*PHOSPHATE TRANSPORTER1;4*, *PHT1;4*) (Supplementary Table 5). Furthermore, catalase-encoding genes within the dark green module (*slb1 S*2-specific) were assigned to the GO term “Response to oxidative stress” (Fig. 6b; Supplementary Table 5) and the KEGG pathway “MAPK signaling” (Supplementary Fig. 9, denoted by red underlined). All these genes had the highest expression level in the S2 stage of *slb1* (Supplementary Table 5), suggesting a potential role of nutrient or oxidative cues specific to lateral bud (Fig. 3a, yellow arrow) activation and outgrowth of *slb1* mutants. (Complete results of GO enrichment and KEGG pathway analyses are shown in Supplementary Figs. 7 and 8, respectively).

### Transcriptomic differentiation of plant hormone genes related to shoot branching

Considering the abnormal continuous outgrowth of axillary buds after the normal flowering period in *slb1* mutants, key plant hormone signaling pathways involved in shoot branching, such as auxin, cytokinin and strigolactone (SL) signaling, might be deregulated in these mutants. We therefore examined the expression profiles of key components involved in the biosynthesis, transport and signaling of these hormones between WT and *slb1* plants.

**Fig. 7:**
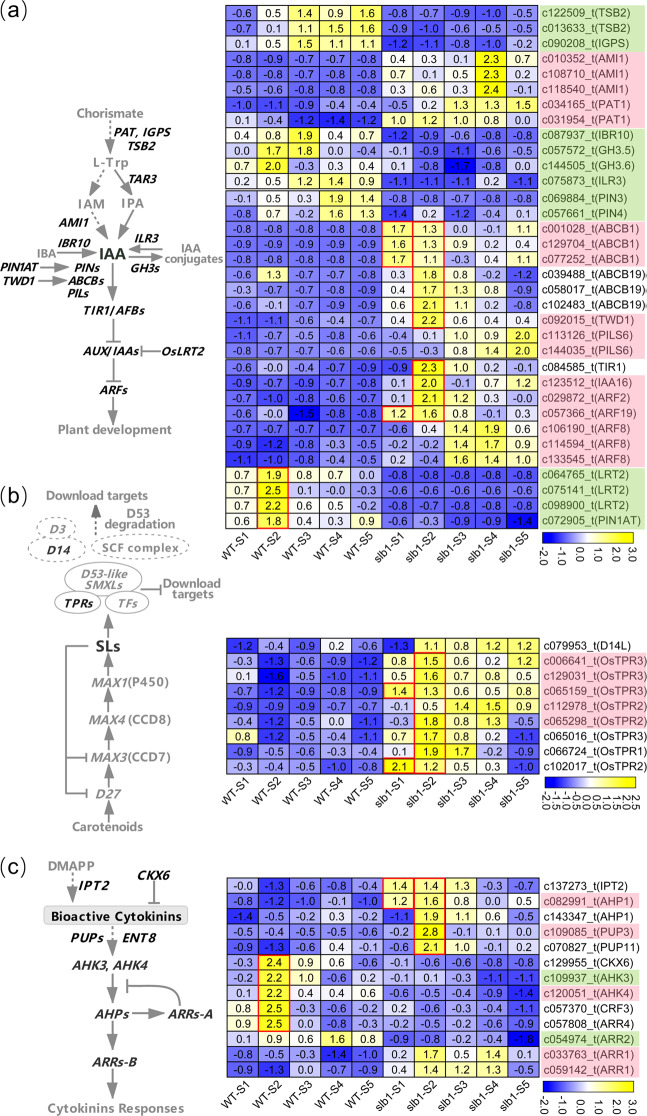
Schematics of biosynthesis, inactivation, transport and signaling and differential transcript profiles of hormone-related genes. **a** Auxin, **b** strigolactone, **c** cytokinin. Genes up- or downregulated in all stages in *slb1* are denoted by light red and green, respectively. Unmarked genes were up- or downregulated in most of the five stages in *slb1*

A key step in the auxin biosynthetic pathway is the conversion of chorismite into L-tryptophan (L-Trp) by three enzymes, namely, PHOSPHORIBOSYLANTHRANILATE TRANSFERASE 1 (PAT1), INDOLE-3-GLYCEROL-PHOSPHATE SYNTHASE (IGPS), and TRYPTOPHAN SYNTHASE BETA-SUBUNIT 2 (TSB2). L-Trp can then be further converted into indole-3-acetic acid (IAA), a biologically active auxin, by the enzyme AMI1. We identified in *L. chinense* two genes that may encode PAT1 (c034165_t and c031954_t) and three genes encoding AMI1 (c010352_t, c108710_t, and c118540_t) that were highly expressed in *slb1* (Fig. 7a). In contrast, homologs of the *IGPS* (c090208_t) and *TSB2* (c122509_t and c013633_t) genes were relatively highly upregulated in the WT, particularly in the S3-S5 stages (Fig. 7a). In addition, *INDOLE-3-BUTYRIC ACID RESPONSE 10* (*IBR10*) (c087937_t), which functions in the conversion of the auxin precursor indole-3-butyric acid (IBA) to IAA, was relatively highly expressed, especially in the WT-S3 stage. Furthermore, *GRETCHEN HAGENs* (*GH3s*) (c057572_t and c144505_t) and *IAA-LEUCINE RESISTANT3* (*ILR3*) (c075873_t), promoting and restraining the synthesis of IAA conjugates, respectively, were relatively highly expressed in the WT in the S1-S3 stages and S3-S5 stages, respectively (Fig. 7a).

We found that auxin transporters of the B-type ATP-binding cassette (ABCB) family, ABCB1 (c001028_t, c129704_t, and c077252_t) and ABCB19 (c039488_t and c058017_t) as well as the ABCB chaperone *TWISTED DWARF1* (*TWD1*)^29^ (c102483_t), were highly expressed in the S1 and/or S2 stage in *slb1* mutants (Fig. 7a). In addition, we also found that members of the classical “TIR1/AFB-AUX/IAA-ARFs” auxin signaling pathway, including *TRANSPORT INHIBITOR RESPONSE 1* (*TIR1*) (c084585_t), *INDOLEACETIC ACID-INDUCED PROTEIN 16* (*IAA16*) (c123512_t), *AUXIN RESPONSE FACTOR*s (*ARFs*) *ARF2* (c029872_t), and *ARF19* (c057366_t), were all especially highly expressed in the S2 stage in *slb1* mutants (Fig. 7a). In addition, *ARF8* (c106190_t, c114594_t, and c133545_t) was also upregulated in *slb1* mutants, with the expression peak occurring in the S3-S5 stages instead of the early stages (Fig. 7a). In addition, *LATERAL ROOTLESS2* (*LRT2*) (c064765_t, c075141_t, and c098900_t) and *PIN1-type parvulin 1* (*PIN1AT*) (c072905), known to influence auxin signaling in rice^30^ and auxin transport in *A. thaliana*^31^, respectively, were especially highly expressed in the S2 stage in WT plants (Fig. 7a).

*IPT2* (c137273_t), encoding ISOPENTENYL TRANSFERASE, involved in cytokinin biosynthesis, was upregulated in the S1-S3 stages, whereas *CKX6* (c129955_t), encoding CYTOKININ OXIDASE, involved in cytokinin conjugation, was downregulated in the S2-S4 stages in WT plants (Fig. 7c). Two members of the purine permease (PUP) family, which has been implicated in the influx transport of cytokinins^32^, (c109085_t and c070827_t), were most highly expressed in the S2 stage in *slb1* mutants (Fig. 7c). In addition, *ARABIDOPSIS HISTIDINE PROTEIN 1* (*AHP1*) (c143347_t), one of the phosphor-transfer intermediates for AHK-AHP-A/B-ARR modules in the cytokinin signaling pathway, was also relatively highly expressed in the S2 stage in *slb1* mutants (Fig. 7c). Furthermore, two A-type *ARABIDOPSIS RESPONSE REGULATORS* (*ARRs*), *ARR2* (c054974_t) and *ARR4* (c057808_t), which negatively regulate cytokinin signaling, were relatively highly expressed in the S2 stage in WT plants (Fig. 7c). In contrast, two B-type *ARRs*, *ARR1* (c033763_t and c059142_t), which positively regulate the cytokinin signaling pathway, were relatively highly expressed in the S2-S4 stages in *slb1* mutants (Fig. 7c).

In addition, DWARF-14-LIKE (*D14L*) (c079953_t), a gene that functions in the response to karrikins, a class of butenolide compounds structurally related to SL^33^, was relatively highly expressed in the S2-S5 stages in *slb1* mutants (Fig. 7b). Importantly, *TPRs* (c006641_t, c129031_t, c065159_t, c065016_t, c066724_t, c065298_t, c102017_t, and c112978_t), encoding TOPLESS-related transcriptional corepressors that are known to be involved in branch formation downstream of SL signaling^33^, showed high expression in *slb1* mutants, especially in the S2 stage (Fig. 7b).

Together, these results indicate extensive transcriptional differences in components related to auxins, cytokinin and SL, particularly in the S1 and/or S2 stage, between *slb1* and WT plants.

### Differential expression of genes related to flowering timing and flower development regulation

**Fig. 8:**
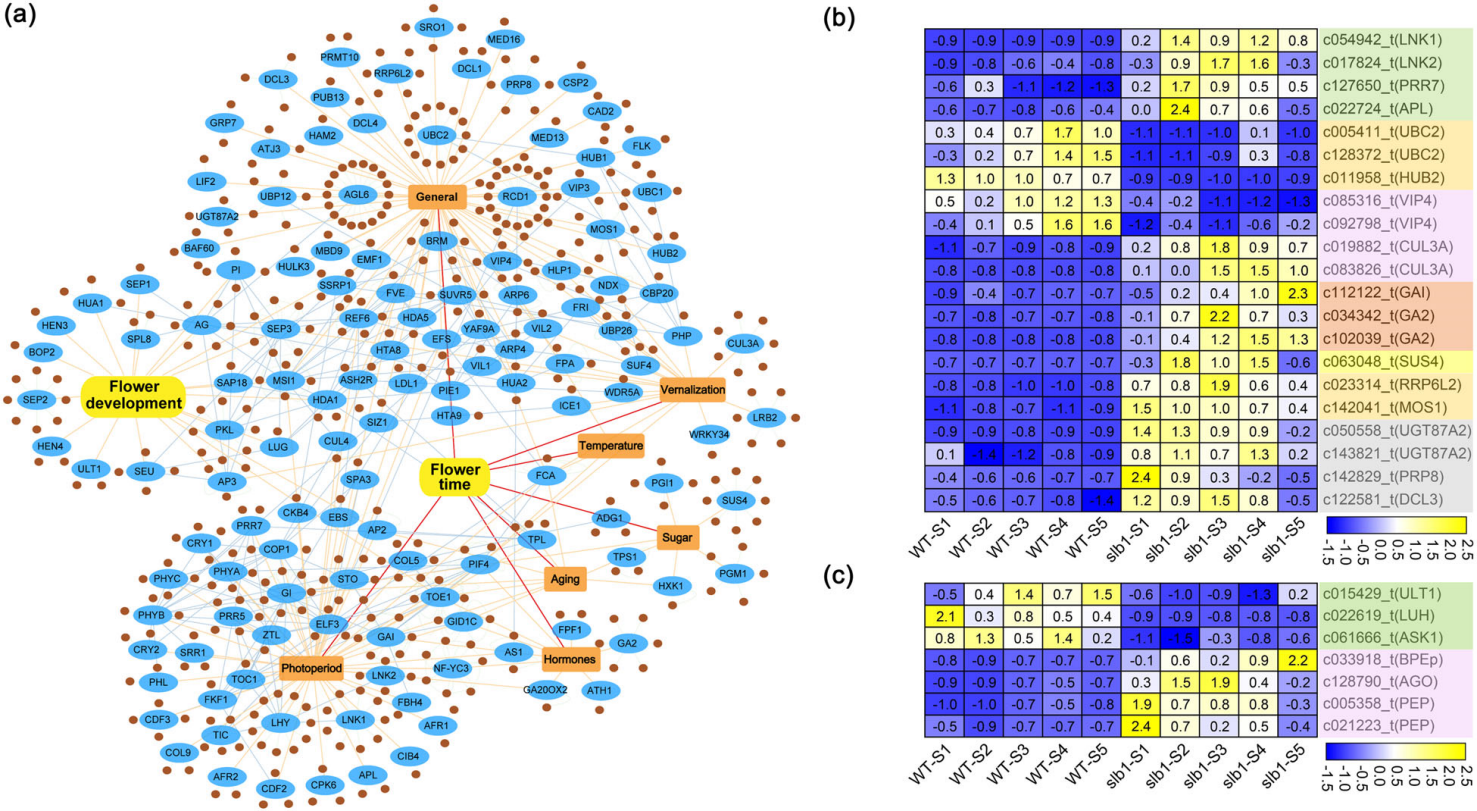
Genes related to flowering timing and flower development. **a** Flowering-related genes identified in *slb1* mutants of *L. chinense* (brown circle) and their corresponding homologous genes in Arabidopsis (blue ellipse). Blue lines show experimentally determined gene interactions obtained from the String (https://string-db.org/) database. **b**, **c** Heatmap showing the expression profiles of genes related to flower timing and flower development regulation. **b** Heatmap showing the expression profiles of genes related to photoperiod (light green), vernalization (pink), gibberellin (brown), sugar (yellow), temperature (light blue), epigenetic (light golden), and general and autonomous (gray) flowering pathways. **c** Heatmap depiction of transcriptional differences of flower development-related genes identified in the FLOR-ID database (light green) and previous publications (pink)

To gain a comprehensive view of the gene networks and their potential roles in flowering time and flower development in the *slb1* mutant, we used the FLOR-ID database^3^ as a source of genes known to be involved in flowering timing and flower development in plants and found a total of 543 homologous genes predicted to be involved in flowering regulation in *Liriodendron* (Fig. 8a). These genes are part of a complex and integrated network of multiple pathways, including components that mediate responses to photoperiod, light quality, temperature, and age; hormone biosynthesis and signaling; sugar level and epigenetic status (Fig. 8a), enabling and regulating the flowering transition or flower development.

Of these, environment-response factors, i.e., *NIGHT LIGHT-INDUCIBLE AND CLOCK-REGULATED 1*/*2* (*LNK1*/*2*), *PSEUDO-RESPONSE REGULATOR 7* (*PRR7*), and *ALTERED PHLOEM DEVELOPMENT* (*APL*), are involved in photoperiod regulation; the key vernalization-related regulators *VERNALIZATION INDEPENDENCE 4* (*VIP4*), *Cullin-3A* (*CUL3A*), and *PHYTOCHROME INTERACTING FACTOR 4* (*PIF4*) are involved in the ambient temperature pathway. The GA biosynthetic pathway component *GA REQUIRING* 2 (*GA2*) and GA signaling-related genes, including *GIBBERELLIC ACID INSENSITIVE* (*GAI*), as well as the sucrose synthase-coding gene *SUCROSE SYNTHASE 4* (*SUS4*), which act as endogenous cues in flowering control, were also identified. In addition, several known epigenetic regulator-related genes were detected, including *UBIQUITIN-CONJUGATING ENZYME 2* (*UBC2*), HISTONE MONO-UBIQUITINATION 2 (*HUB2*), *MODIFIER OF SNC1* (*MOS1*), and *RRP6-LIKE 2* (*RRP6L2*). Using our RNA-seq data, we found that all these genes, which positively or negatively regulate flowering time in *A. thaliana*, were highly or weakly expressed in *slb1* and may promote *slb1* flowering (Fig. 8b). This potentially contributes to the difference in flowering time between *slb1* and WT plants.

In addition, *ULTRAPETALA1* (*ULT1*), *PICKLE* (*PKL*), and several other genes (*LEUNIG_HOMOLOG* (*LUH*), *ASKalpha* (*ASK1*), *BIG PETAL P* (*BPEp*), *ARGONAUTE 1* (*AGO1*), and *PEPPER* (*PEP*)) functioning in flower development and meristem identity^3,34–38^ were differentially expressed during flower bud development between *slb1* mutants and WT plants (Fig. 8c), suggesting that they participate in floral homeostasis in *slb1* mutants.

### Quantitative real-time PCR (qRT-PCR) assays of DEGs

We performed qRT-PCR analysis to measure the relative transcript levels of 7 representative DEGs, including 2 auxin transport-related genes (the *ABCBs* c129704_t and c102483_t) (Fig. 7a) and 5 flowering timing- or flower development-related genes, namely, *SUS4* (c063048_t) and *ASK1* (c061666_t) (Fig. 8), *SEP3* (c012890_t) (Fig. 4d), and *FT* (c084733_t and c026545_t) genes (Fig. 5). Most of the tested genes showed similar expression profiles to those in the RNA-seq data (Supplementary Fig. 10b), confirming a high correlation between the RNA‐seq and qRT-PCR data (Supplementary Fig. 10c).

## Discussion

### Indeterminate shoot growth in *slb1* mutants of *L. chinense*

Plants have evolved species-specific visual appearances and structural features to enable them to optimize their adaptation to the environment and reproductive strategies. A plant’s growth habit is determined by the pattern of vegetative organs and reproductive structures arranged along the shoot axes and by the organization of lateral branches^39^. In *Liriodendron*, the production of a flower on the shoot apex (Fig. 1c) marks the cessation of main shoot growth, and growth continues from secondary branches out of axillary buds. This growth pattern is referred to as “sympodial” in perennial plants. We noted modified branching patterns along the stem in *slb1* mutants of *L. chinense*. With the advent of spring, the dormant *slb1* flower buds, located at the shoot apex, are activated and develop towards maturity. Meanwhile, subapical axillary buds in the uppermost leaf axils, as well as apex buds of a number of annual or biennial branches, initiate a rapid growth cycle; subsequently, these newly formed shoots eventually transform into flowers, usually after producing 2-9 stem nodes. Once the first terminal flowers form, the lateral axillary bud beneath the terminal flower will become the new growth center and rapidly develop into secondary flowering shoots terminated by a single flower (Fig. 1f). The secondary flowering branches themselves repeatedly produce secondary flowering branches, and reiteration of this process results in recurrent cycles consisting of vegetative and floral phases (Fig. 1f, k). The primary shoot is always replaced by newly outgrown side shoots, thus making the *slb1* mutant stem seemingly upright and continuous (Fig. 1f, k).

A similar form of branching was reported in wild tomatoes. The tomato plant is a compound shoot system made up of multiple sympodial shoot units, each of which consists of three leaves and a zigzag-patterned terminal inflorescence^39,40^ (Supplementary Fig. 11). Every sympodial unit renews from the nearest node of the earlier arising unit, and these successively arising units are arranged seemingly upright along the sympodial shoot^39^ (Supplementary Fig. 11). The growth habit of wild tomatoes is classified as “indeterminate” due to the successive formation of an indefinite number of determinate sympodial units^39^. Therefore, the observation of indefinitely extended flower shoots originating from recurrent lateral branches suggests a new, indeterminate growth habit of flowering shoots in *slb1* mutants of *L. chinense*.

### Continuous branching from lateral buds in *slb1* mutants of *L. chinense*

Branching in perennials must be synchronized with seasonal environmental changes by coordinated cycles of growth and dormancy^41^. Prior to the cold winter, bud growth is arrested by dormancy, while dormant buds are released and subsequent growth resumes when the warmer spring arrives^41,42^. In once-flowering WT plants of *L. chinense*, it normally takes three years for vegetative axillary buds to develop into flower buds and finally bloom. In other words, the outgrowth of the newly formed axillary buds is always suppressed or retarded after bud formation in WT plants. Since this process lasts a long time, dormancy is established twice during the first two winters in three years to help coordinate the seasons; in this way, the newly developing shoots can avoid fatal damage. In contrast, accelerated temporal progression of flower branching patterns was observed in *slb1* mutants of *L. chinense*. After the formation of lateral buds, the most proximal axillary bud of the floral apex undergoes immediate outgrowth and rapidly develops into a secondary flowering shoot terminated by a single flower (Fig. 1f, i, k). That is, the outgrowth of lateral buds and subsequent maturation of lateral shoots is accelerated greatly, leading to a much shorter period during which the process of flower initiation and transition is completed in *slb1* mutants. Therefore, *slb1* mutants bloom continuously via the release of lateral buds, during which floral transition and floral development are well established, leading to precocious reproductive maturation (Fig. 1f, i). Intriguingly, early flowering was observed in the sexual progeny of *slb1* mutants at the age of 4 months (Supplementary Fig. 12), whereas it normally takes 8–10 years in WT plants. Thus, we propose that causal molecular mechanisms underlying the continuous flowering behavior in *slb1* mutants of *L. chinense* function in the acceleration of shoot maturation, not only the precocious maturation of lateral buds in adult plants but also the precocious phase transition from vegetative to reproductive growth in seedlings.

Shoot branching is mostly determined by the formation and subsequent outgrowth of axillary buds in leaf axils. We found that members of the *DRM1*/*ARP* (*DORMANCY ASSOCIATED GENE-1*/*AUXIN-REPRESSED PROTEIN*) gene family, which are mainly expressed in dormant, nongrowing or mature tissues and are considered markers of dormancy^43,44^, are highly expressed in mixed buds with arrested lateral buds (S1 and S2 stages, Fig. 3a, red arrow) and almost mature flower buds (S5 stage, Fig. 3a) in WT plants (Supplementary Fig. 13). The highest expression level appeared in the autumn mixed bud stage (S1 stage) (Supplementary Fig. 13), possibly due to its response to the coming winter for dormancy preparation, in addition to the existence of the nongrowing lateral bud (Fig. 3a, red arrow). In contrast, these two genes were expressed at very low levels in all stages of *slb1* mutants (Supplementary Fig. 13), indicating a nondormant phenotype of lateral axillary buds in the *slb1* mutants (Fig. 3a, yellow arrow).

The network of interacting auxin, cytokinin, and SL signals affects the activity of axillary buds^45,46^. The altered branching pattern of *slb1* mutants can be explained by the fact that the formation of terminal flowers releases apical dominance, allowing axillary buds at the upper nodes to develop. Auxin has long been central to apical dominance control^47^. A model of auxin affecting bud outgrowth suggests that auxin flow from axillary buds is competitively inhibited by auxin from the stem^48^ and that the export of auxin from the axillary bud is essential for sustained bud outgrowth^46,48^. In our results, auxin efflux carrier *ABCBs*, as revealed by RNA-seq (Fig. 7a) and qRT-PCR (Supplementary Fig. 10b), were especially highly expressed in the S1 and/or S2 stage in *slb1* mutants. Similar expression profiles were noted in the positive regulator of ABCB-mediated auxin transport, *TWD1*^29^; members of the auxin signaling pathway (*TIR1*, *IAA16*, and *ARFs*); the genes *IPT2* and *PUPs*, which are involved in cytokinin biosynthesis and transport, respectively (Fig. 7c); and *TPRs* (Fig. 7b), putative regulators of SL signaling and mediators of branch formation. These differentially expressed hormone components related to branching display a strikingly consistent expression pattern, with the highest expression levels in the *slb1*-S1 and/or *slb1*-S2 stages, corresponding to the difference in rapid growth of later buds (Fig. 3a, yellow arrow) in the mixed buds of *slb1* mutants compared to the nongrowing status (Fig. 3a, red arrow) in WT plants. Therefore, it is reasonable to presume a role for activators of these hormone components in *slb1* lateral bud development.

### *FT* splicing provides a potential target for the regulation of distinct flowering behaviors in *slb1* mutants

The phenotype of continuous flowering in perennials has previously been reported to be associated with the flowering repressors *FLC* and *TFL1*. Specifically, *Arabis alpina* with a mutation in *PEP1* (ortholog of Arabidopsis *FLC*) flowered continuously for at least 12 months^15^. However, no *FLC* homolog has been identified in the *Liriodendron* genome (Supplementary Fig. 5), consistent with previous studies showing that it is difficult to identify *FLC*-like genes based on homology searches^28,49^. Mutation of orthologs of *TFL1* causes continuous flowering in rose and *Fragaria vesca*^14^, while silencing of *TFL1* in apple and pear leads to precocious flowering^50,51^. Although a putative *TFL1* ortholog (LICH19G1141) has been identified in the *Liriodendron* genome (Fig. 5a), no transcript was detected in our data.

As a flowering integrator, *SOC1* plays an important role in regulating flowering time^23^. We detected two transcripts of *SOC1* in *Liriodendron*, both of which were highly expressed in Phase I and expressed at low levels during flower development in both *slb1* mutants and WT plants (Fig. 4a), suggesting an important role of *LcSOC1* in the floral transition but not in the determination of continuous flowering in *slb1* mutants. *FT*, another core node in multiple flowering pathways, is transcriptionally activated in leaves, and subsequently, FT protein is transformed to the shoot apex, where it forms a complex with FD, promoting flowering^52^. We found that the transcript with the normal splicing variant possessed similar expression patterns between *slb1* mutants and WT plants, while the other transcript contained an intron retention, specifically in Phase II, as verified by RT-PCR (Fig. 5e, f) and qRT-PCR (Supplementary Fig. 10b), in *slb1* mutants.

Cases of alternative splicing of *FT* have been reported in *Brachypodium*^53^ and coconut^54^. In *Brachypodium*, alternative splicing of *FT2* generates two splice variants, *FT2α* and *FT2β*, and the latter cannot interact with FD but can form heterodimers with the former and *FT1* to prevent floral initiation^53^. In coconut, a shorter alternative splice variant of *FT* was extensively present in the dwarf varieties, which need fewer years to begin blooming than the tall varieties in which no shorter variant of *FT* has been found^54^.

In both of these cases, the alternative splice variant of *FT* was missing nucleotides in multiples of three, causing no substantial changes in the protein sequence except for the absence of several amino acids. However, the abnormal *FT* transcript found in *slb1* mutants possessed an additional exon (Fig. 5c), possibly resulting from intron retention, leading to a diverged 3’-end and a truncated protein at a premature stop codon (Fig. 5d). Nevertheless, the 5′-end of this abnormal *FT* variant was identical to that in the normal *FT* transcript, indicating that the protein encoded by this variant might still be at least partially functional. Overall, this abnormal splicing variant of *FT* offers a potential target for dissecting the genetic control of continuous flowering in *slb1* mutants of *L. chinense* in future research.

### Potential value of *slb1* mutants of *L. chinense* in genetics and breeding

A longstanding challenge impeding the genetic improvement of perennial trees is their long maturation period, known as the juvenile phase, before they reach reproductive maturity. As described above, progeny of *slb1* mutants of *L. chinense* flowered precociously at a seedling age of 4 months. This rare natural variant provides the possibility to develop an effective transgenic receptor system with a short juvenile period and may even serve as a model plant for genetic studies in woody species. It can be handled easily within the laboratory, as it flowers at an age when its size is still small (Supplementary Fig. 12). We have confirmed that *slb1* mutants are self-fertile by artificial pollination across multiple seasons. Their flowering over a long time span will increase the possibility that male and female gametophytes experience different environmental conditions (e.g., high ambient temperatures), possibly disturbing their development, which may help produce some positive trait variations with breeding value. These strongly prolonged flowering *slb1* mutants of *L. chinense* have potential agronomic value as precious germplasms. The availability of such *L. chinense* variants might provide new breeding lines suitable as ornamental plants, apart from the typical application in landscaping and forestation as large deciduous trees.

## Materials and methods

### Flower morphology observation

All *slb1* mutant plants and WT plants of *L. chinense* used in this work were grown in the *Liriodendron* germplasm nursery of Nanjing Forestry University. The numbers of tepals, stamens and pistils per flower were scored once every one or two weeks, and a headband magnifier or stereomicroscope was applied for pistil number counting when necessary. This observation was carried out during full blooming, starting in April and extending through October. The number of floral organs was determined for more than 240 *slb1* flowers.

### Histological sectioning of mixed buds

For cytological studies, 5 buds were collected each time (late February to early November 2016; once a week) and fixed in 50% FAA. They were then dehydrated using a graded ethanol series and embedded in paraffin (Sigma). Eight-micrometer-thick sections were cut with a Leica RM2145 rotary microtome and stained with Safranin O and Fast Green for viewing under a Zeiss microscope. Images were photographed using the Zeiss Axio Vert. A1 system.

### Transcriptome sequencing and data analysis

Total RNA was extracted from samples taken at five developmental time points (each with three biological replicates) from both WT and *slb1* mutant plants, including mixed buds (autumn bud, S1, and spring bud, S2, sampled in early September and June, respectively, with each biological replicate consisting of 2 buds) and flower buds at three successive but distinctive developmental phases (S3-S5, sampled in early May; only one bud was collected for each biological replicate) (Fig. 3a). RNA samples from mixed buds (S1 and S2) and flower buds (S3–S5) were prepared for PacBio Iso-Seq. All sampled tissues were quickly frozen in liquid nitrogen and stored at –80 °C until use.

RNA was isolated using the CTAB method and then quantified and quality-assessed using the Agilent Bioanalyzer 2100 system. High-quality RNA was used for library preparation and then sequenced on the PacBio RS and Illumina HiSeq X Ten platforms. The SMRT Link v5.0 package was used for Iso-Seq raw data analysis, and full-length sequences were obtained after further self-correction and clustering. PacBio transcript quality was further improved by employing Illumina short read alignment to assist erroneous correction using Proofread v2.12 software. Unique, nonredundant sequences were generated after a clustering step using CD-HIT_EST v4.7 software. Cleaned reads obtained after removing adapters from raw Illumina data by Trimmomatic v0.36 were mapped to the reference transcript sequence using Rsem v1.3.0 to measure the number of mapped reads per transcript. Differential expression analysis based on the data of three biological replicates between two groups was performed using DESeq2 v3.6. For functional prediction, unigenes were annotated based on public databases: Swiss-Prot, KOG, GO and KEGG. Identified homologs of Arabidopsis genes were used for transcription factor searches in the PlantTFDB (5.0) database (http://planttfdb.cbi.pku.edu.cn/). GO and KEGG enrichment and visualization were conducted using the ClusterProfile R package. The WGCNA v1.68 R package was applied for coexpression network construction. UpSet plots and heatmaps were created using the TBtools toolkit (https://www.biorxiv.org/content/10.1101/289660v1).

### qRT-PCR analysis

The same samples for RNA sequencing were used as the source of RNA for qRT-PCR. Data were derived from three biological replicates and calculated with the Pfaffl method. The actin^55^ and ubiquitin genes were used as reference genes. The primer sequences and qRT-PCR programs are listed in Supplementary Table 6.

### Detection of the two splice variants of *FT*

Specific primer sequences and PCR programs for amplification of alternative splicing of *FT* are listed in Supplementary Table 7.

## Supplementary information

Supplementary Figure S1-S13

Supplementary Table S1-S7

## Data Availability

Raw reads have been submitted as a BioProject under accession PRJNA677852.
