# Supplementary material for: Morphological, phenological, and transcriptional analyses provide insight into the diverse flowering traits of a mutant of the relic woody plant Liriodendron chinense
Source: Hortic Res. 2021 Aug 1;8:174. doi: 10.1038/s41438-021-00610-2 (PMC8325688; doi:10.1038/s41438-021-00610-2)
Supplement: Supplementary file 1 — Supplementary Figure S1-S13 [file 41438_2021_610_MOESM1_ESM.pdf]

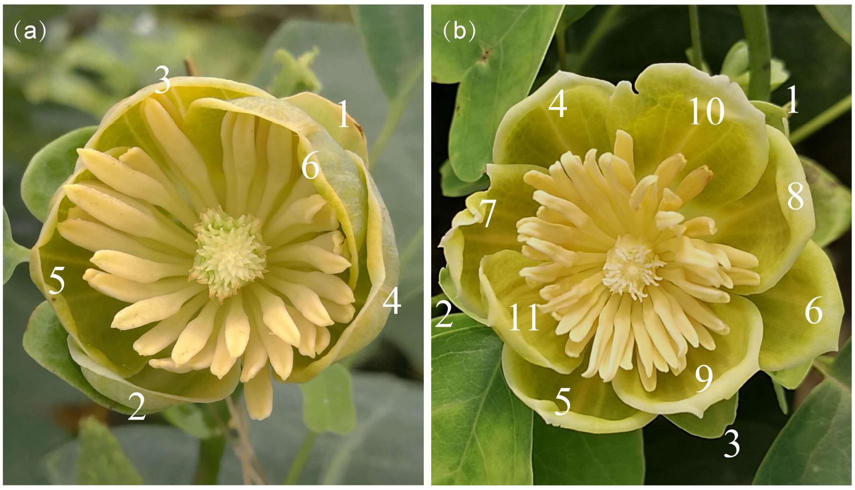

Supplementary Fig. 1 Sporadic presence of *slb1* mutant flowers with 6 or 11 tepals. (a) 6-tepaled *slb1* flowers lose the trimerous and radially symmetric patterns compared to that in WT plants. (b) 11-tepaled *slb1* flowers have extra two tepals arisen inner the three trimerous flower whorls.

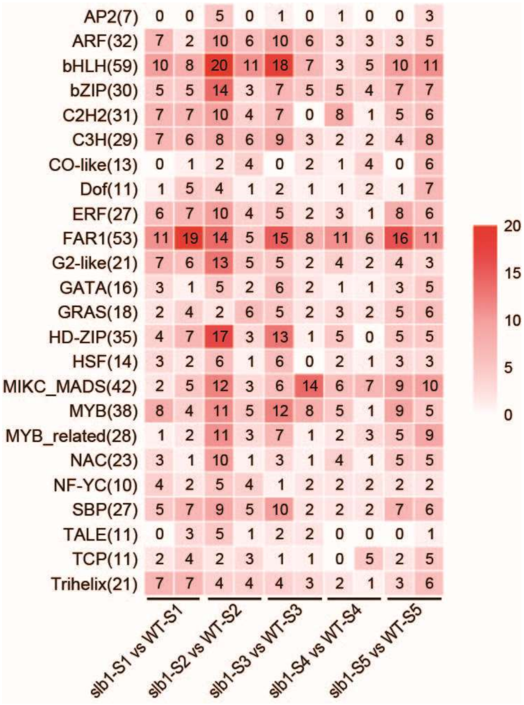

Supplementary Fig. 2 Heatmap distribution of the number of TF genes per TF family up- or down- regulated per pairwise comparison (a total of 5) of successive developmental stages of flower buds between *slb1* and WT genotypes. The sum of TFs number of each family from DEGs in all pairwise comparisons is indicated in parentheses (the same gene within one TF family appears multiple times across different developmental stage comparisons is defined as one); only those TF families with at least 5 members in any pairwise comparison are shown.

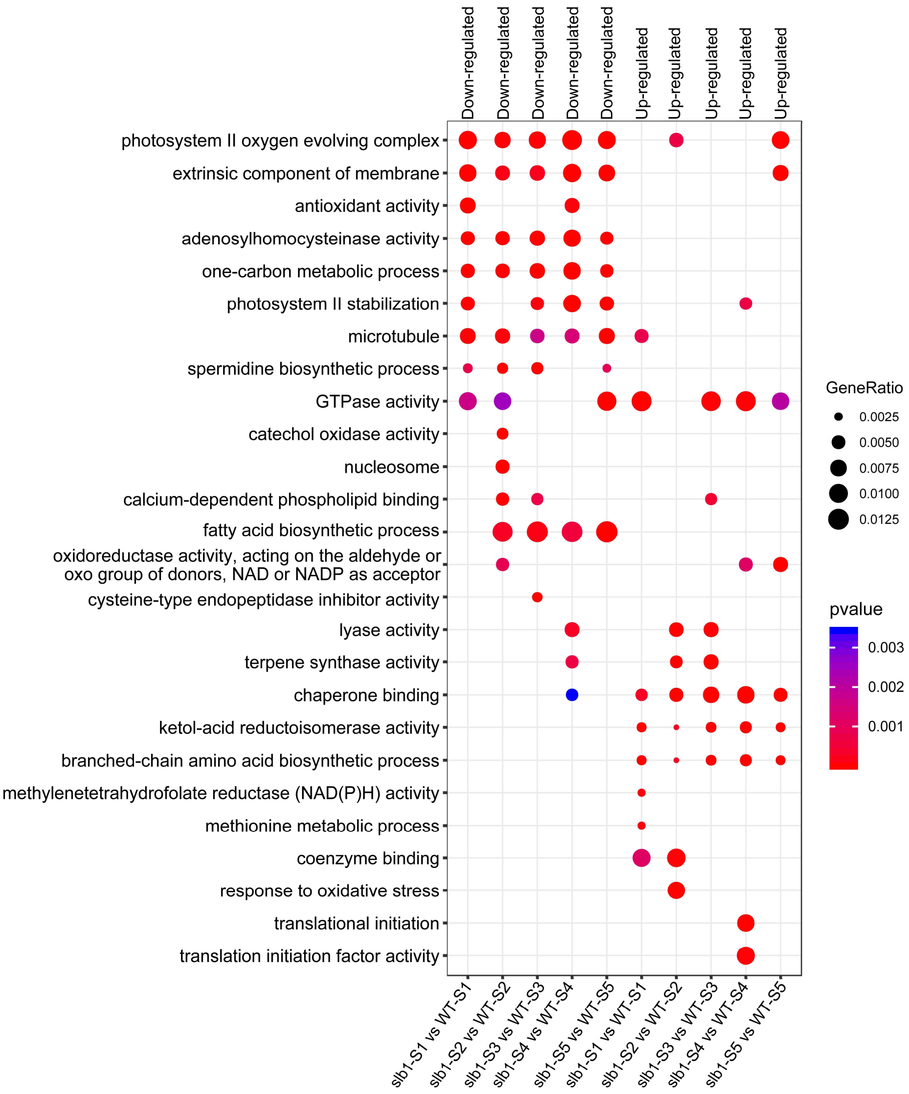

Supplementary Fig. 3 Overlapping GO term analysis of both up-regulated and down-regulated DEGs revealed categories specifically or commonly enriched in one or multiple pairwise comparisons.

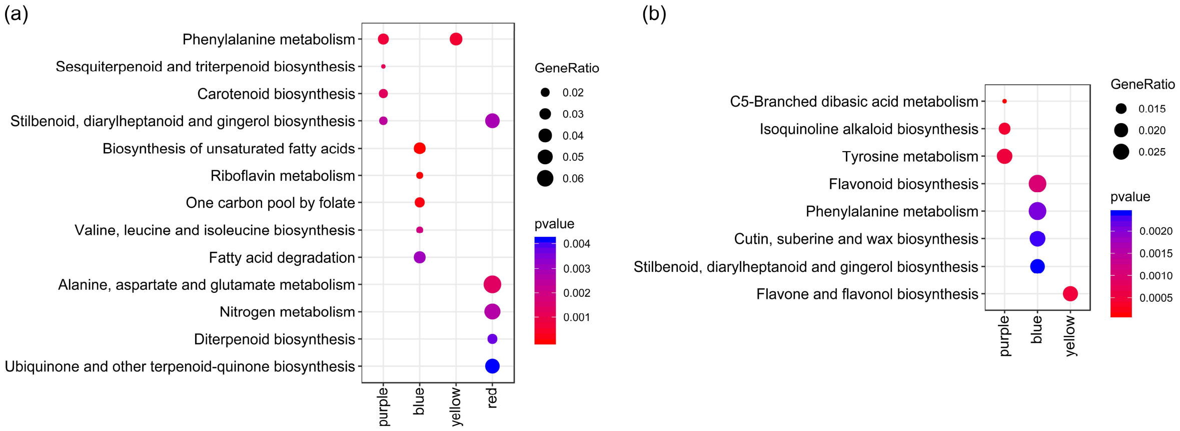

Supplementary Fig. 4 Functional classifications for a part of specific and common up- and down-regulated DEGs obtained via Upset plot. (a) KEGG pathway enrichment of unique or common up-regulated DEGs marked colorfully in Figure 4c. (b) KEGG pathway enrichment of unique or common down-regulated DEGs marked colorfully in Figure 4d.

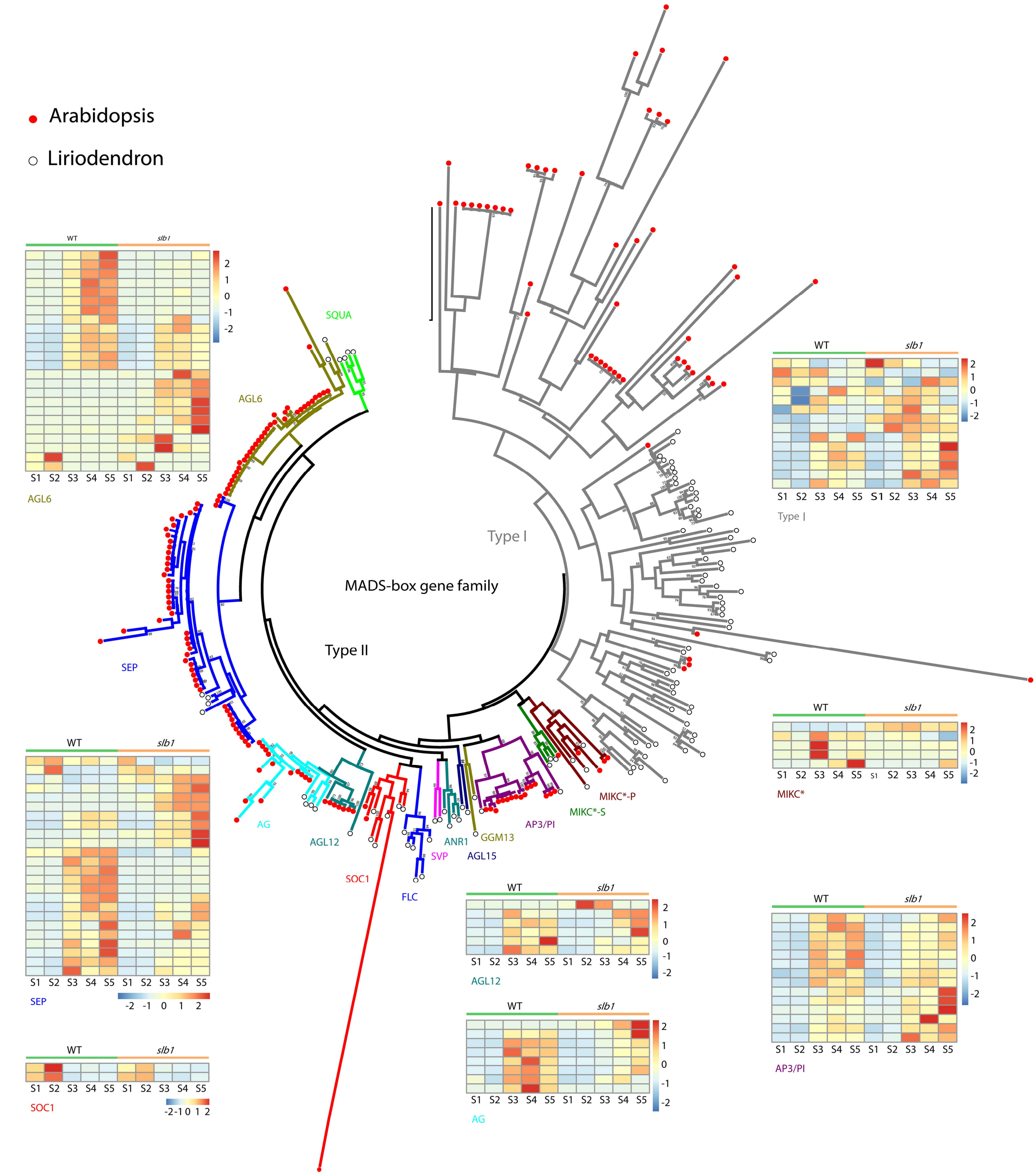

Supplementary Fig. 5 Phylogenetic analysis and expression profiles of MADS-box genes in *slb1* mutants and WT plants. The tree was generated using the ML method based on the amino acid sequences. Different clades are labeled by colors, and MADS-box genes of *Arabidopsis* and *Liriodendron* are marked with red dots and soft dots, respectively.

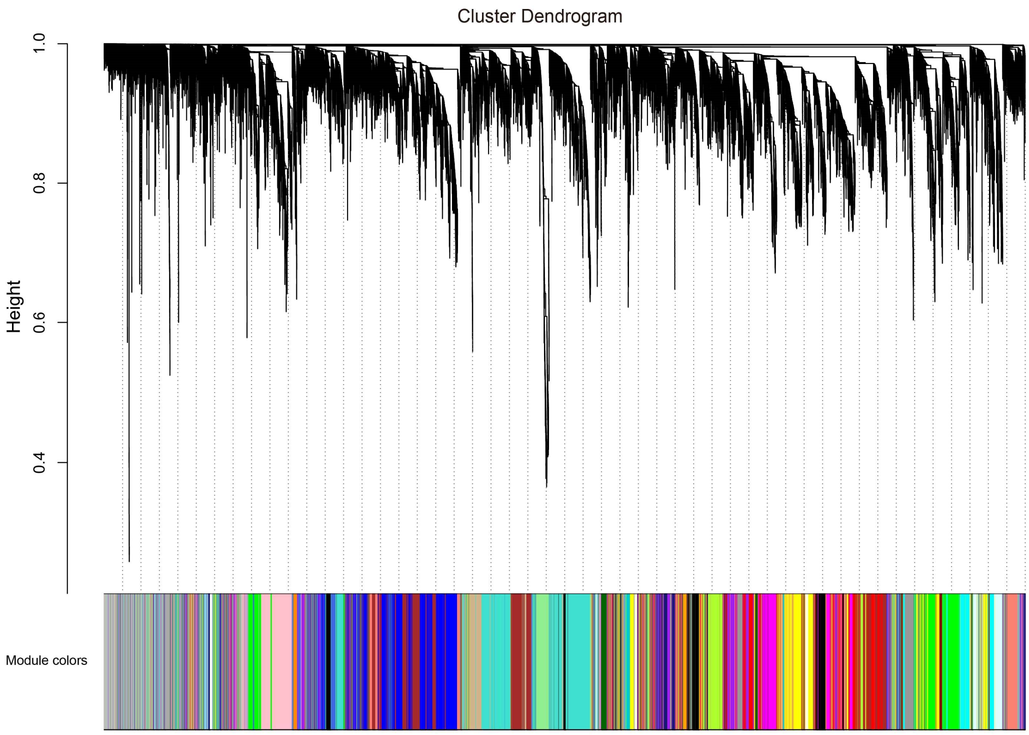

Supplementary Fig. 6 Hierarchical clustering dendrogram showing co-expression modules identified by WGCNA. Each branch (black vertical line) in the dendrogram represents individual gene. Each color of the color rows under the dendrogram corresponds to a module with highly similar expression patterns of genes.

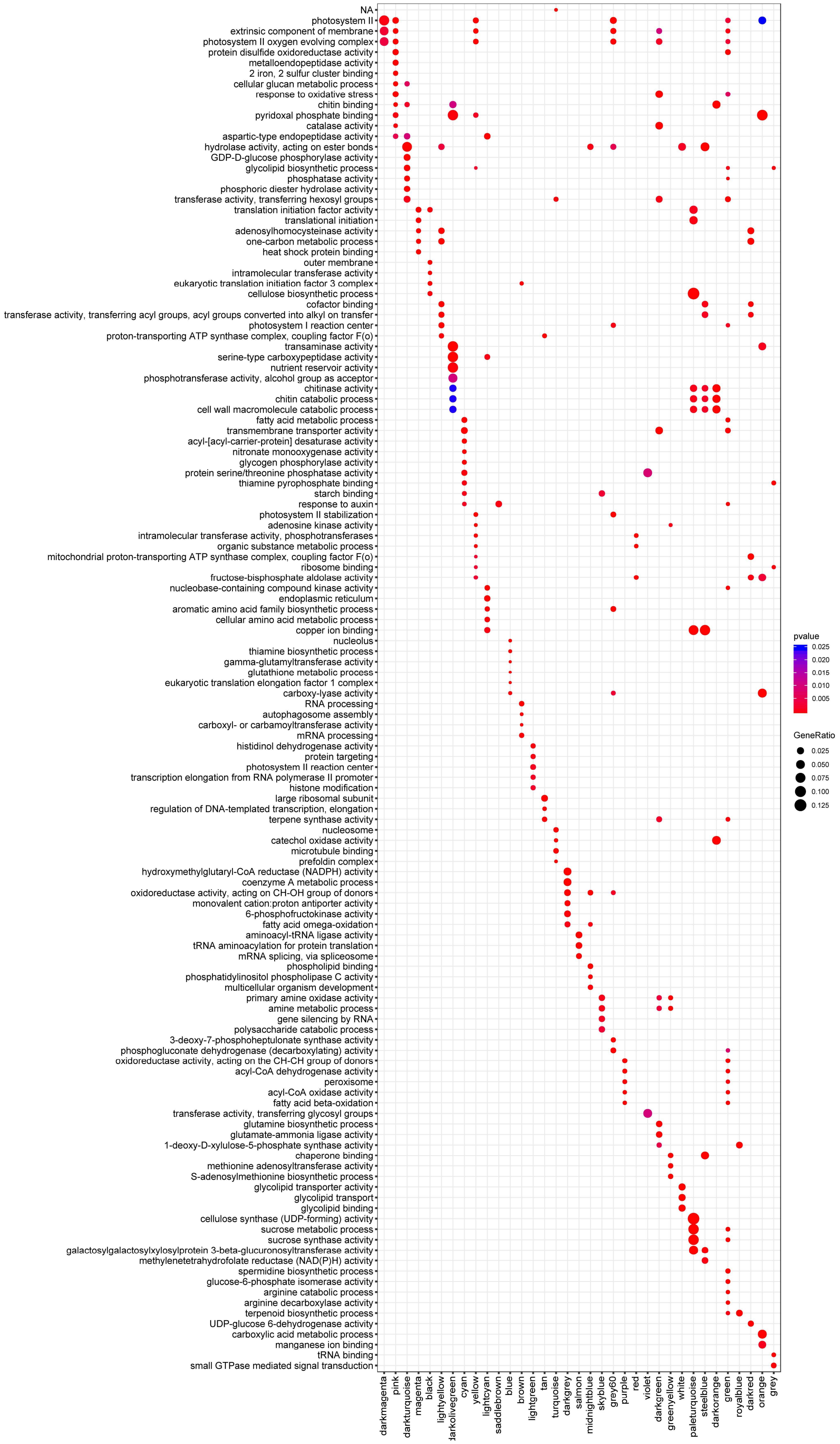

Supplementary Fig. 7 GO enrichment of genes in all WGCNA modules.

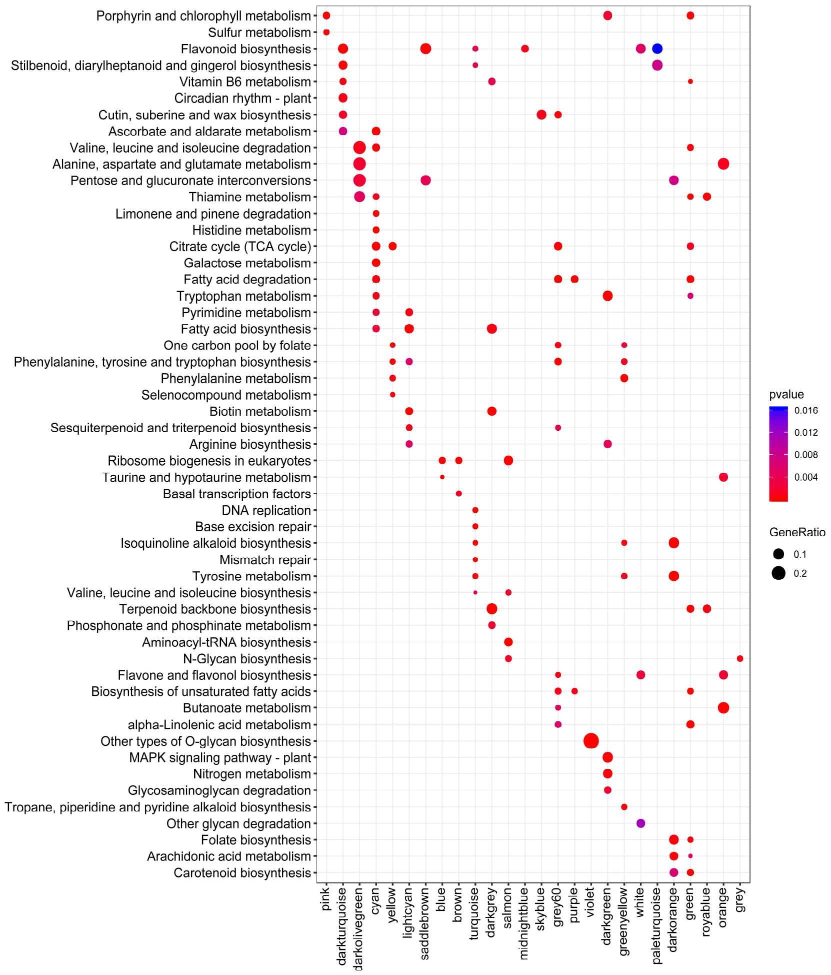

Supplementary Fig. 8 KEGG enrichment of genes in all WGCNA modules.

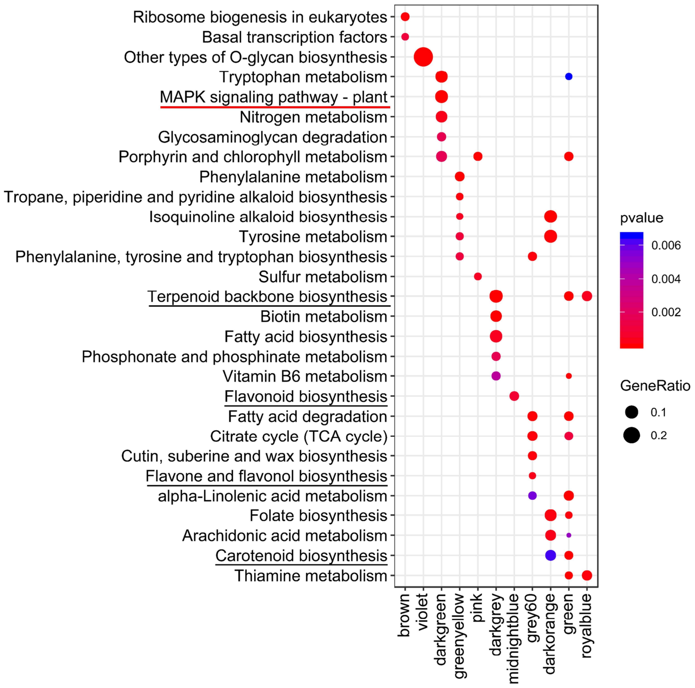

Supplementary Fig. 9 KEGG enrichment of genes in modules that positively correlate with tissue of *slb1* S1 and/or S2, S4 and/or S5.

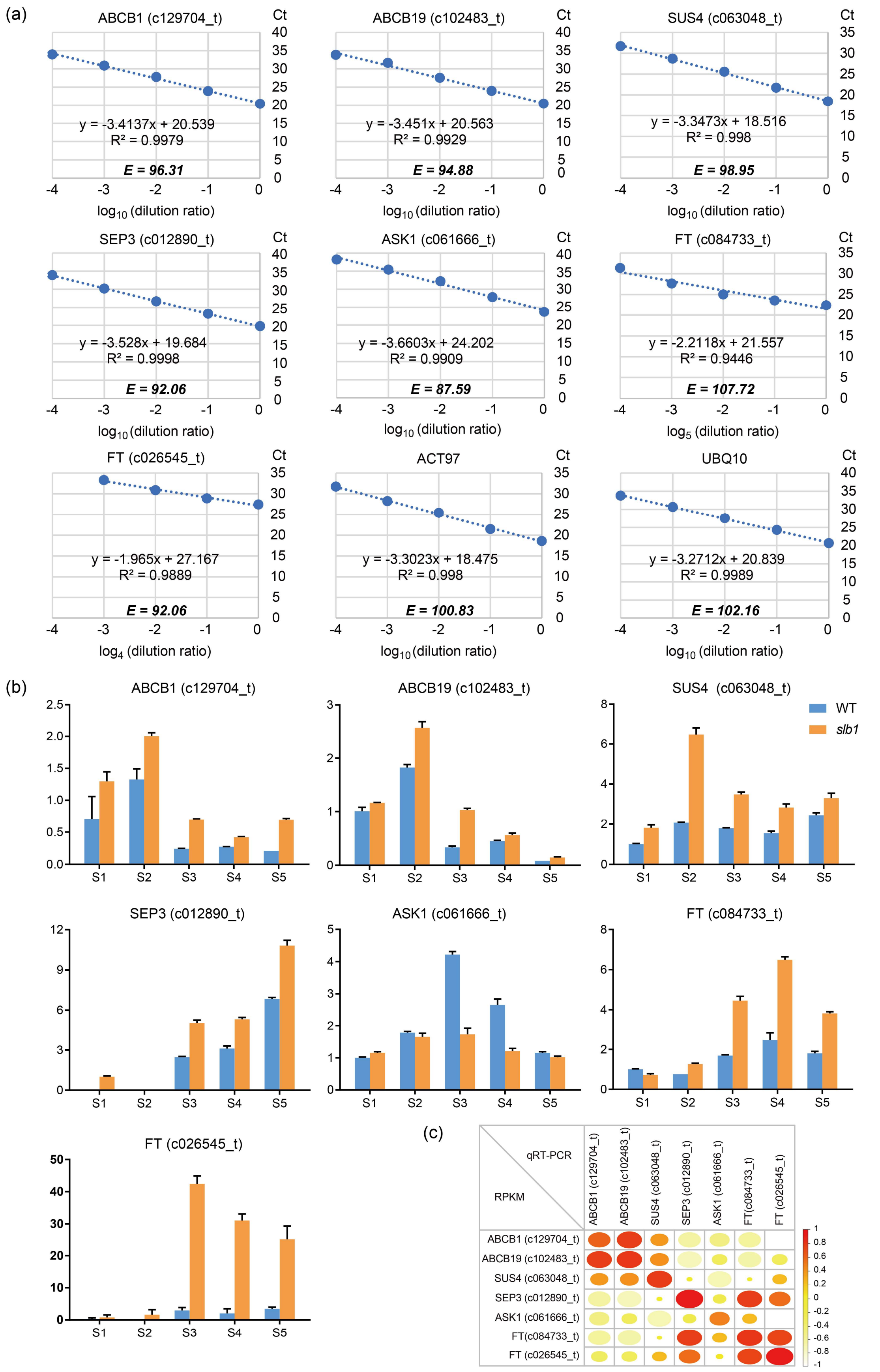

Supplementary Fig. 10 Validation of the DEGs expression by qRT–PCR. (a) Amplification efficiency of qRT-PCR reactions. Primer efficiency (indicated with italics) was measured using 4-, 5- or 10-fold dilutions of the cDNA stock. Curves show log values for the dilution ratio (4 or 5 gradients) plotted against Ct value from qRT-PCR amplification using different primer pairs. (b) Transcript abundance of candidate genes quantified by qRT–PCR. For each gene, the relative expression level was compared to the first stages of wild-type (WT S1). (c) Heatmap shows the correlation between the outputs of RNA-seq and qRT-PCR for the genes tested.

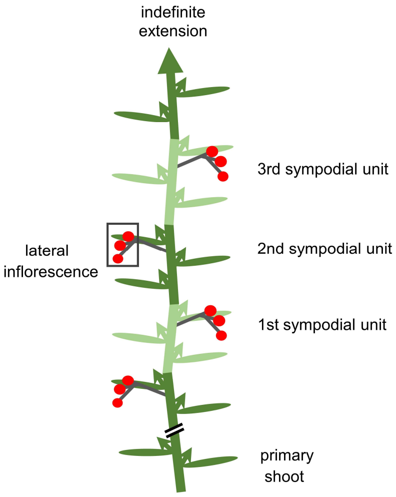

Supplementary Fig. 11 Schematics showing the shoot architecture of indeterminate wild-type tomato, indicating the similar growth habit between *L. chinense slb1* mutant and wild-type tomato. The indefinite number of sympodial segments each consists of three leaves and a zigzag patterned lateral terminal inflorescence arisen repeatedly after every three internodes along the primary shoot (Refering to Pnueli L et al., 1998[32]; Jiang K et al., 2013[33]).

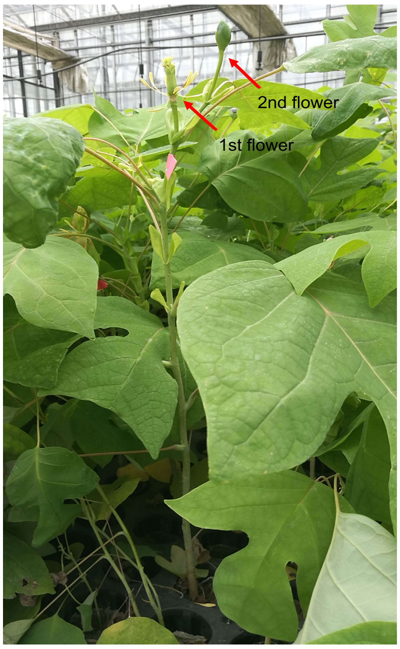

Supplementary Fig. 12 Progeny obtained via sexual reproduction of *slb1* mutants flowered continuously and early at the age of 4 months.

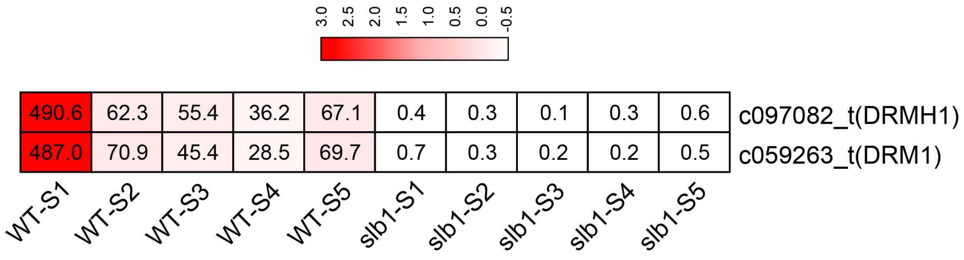

Supplementary Fig. 13 Heatmap showing the expression of two members of *DRM1*/*ARP* gene family that was associated with dormancy. Values represent transcript abundance (average FPKM of three replicates).
