# Supplementary material for: Morphological, phenological, and transcriptional analyses provide insight into the diverse flowering traits of a mutant of the relic woody plant Liriodendron chinense
Source: Hortic Res. 2021 Aug 1;8:174. doi: 10.1038/s41438-021-00610-2 (PMC8325688; doi:10.1038/s41438-021-00610-2)
Supplement: Supplementary file 2 — Supplementary Table S1-S7 [file 41438_2021_610_MOESM2_ESM.pdf]

Supplementary Table 1 Detailed features of *slb1* flowers with 7, 8, 9, 10 tepals. The average number of stamens or pistils of flowers with varied tepals is indicatds in parentheses.

Number of perianths	Number of flower whorls	Number of tepal (s) in each whorl				Number of stamens	Number of pistils
		First	Second	Third	Fourth		
7	3	2	unclear	unclear	—	28~40 (33)	77~136 (108)
8	3	3	2	3	—	27~42 (34)	67~136 (109)
9	3	3	3	3	—	27~48 (40)	87~140 (113)
10	4	3	3	3	1	27~50 (40)	86~143 (120)

Supplementary Table 2 Statistics of PacBio Iso-Seq output data generated from the two SMRT Cells. List of the number of subreads, circular consensus sequences (CCSs), full-length non-chimeric reads (FLNCs) and the percentage of FLNCs (Number of FLNCs/ Total number of CCSs).

Sample	Records of SMRT Cells	Number of subreads	Number of CCSs	Number of FLNCs	Percentage of FLNCs
G1	m54160_171216_174427	5,124,340	644,735	251838	39.06%
F1	m54160_171217_040355	1,856,889	192,193	113613	59.11%

Supplementary Table 3 Summary of full-length non-chimeric reads (FLNCs). List of the information of the FLNCs derived from each workflow including isoform cluster, PacBio self-correction and correction using next-generation sequencing (NGS) reads, and redundancy removing for unigenes identification.

Term	Cluster	PacBio self-correction	Correction using NGS reads	Unigenes
Total number	193,274	193,274	181,937	146,826
Min of length	100	100	270	270
Max of length	11,844	11,844	11,816	11,816
Avergage of length	2,233	2,233	2,377	2,572

Supplementary Table 4 Next-generation sequencing (NGS) reads statistics. Each sample group contains three biological replicates.

File	Group	Number of reads	Number of clean reads	Number of rRNA reads	Percentage of rRNA reads
WT1-1A	WT1	14747697	14445463	100675	0.70%
WT1-2A	WT1	13436584	13141826	58159	0.44%
WT1-3A	WT1	14658618	14344468	92747	0.65%
WT2-1A	WT2	14023208	13871573	37754	0.27%
WT2-2A	WT2	13554200	13409751	55158	0.41%
WT2-3A	WT2	13671978	13533715	53042	0.39%
WT3-1A	WT3	15914080	15536384	48955	0.32%
WT3-2A	WT3	15469306	15095143	48737	0.32%
WT3-3A	WT3	16492627	16097636	59420	0.37%
WT4-1A	WT4	16228679	15837866	47683	0.30%
WT4-2A	WT4	15496934	15132799	77674	0.51%
WT4-3A	WT4	14048151	13710764	28913	0.21%
WT5-1A	WT5	14012975	13684295	63924	0.47%
WT5-2A	WT5	15253862	14917620	71171	0.48%
WT5-3A	WT5	14069740	13709850	56598	0.41%
slb1-1-1A	slb1-1	14389786	14066344	46423	0.33%
slb1-1-2A	slb1-1	13650400	13353406	48311	0.36%
slb1-1-3A	slb1-1	15347115	15023079	47100	0.31%
slb1-2-1A	slb1-2	14403088	14103697	43311	0.31%
slb1-2-2A	slb1-2	17410963	17036871	52253	0.31%
slb1-2-3A	slb1-2	14856868	14525108	38665	0.27%
slb1-3-1A	slb1-3	14275621	13902061	31064	0.22%
slb1-3-2A	slb1-3	14286935	13970558	44871	0.32%
slb1-3-3A	slb1-3	17002949	16593133	70614	0.43%
slb1-4-1A	slb1-4	15772699	15389260	49910	0.32%
slb1-4-2A	slb1-4	15293250	14905421	37108	0.25%
slb1-4-3A	slb1-4	16786155	16373417	33991	0.21%
slb1-5-1A	slb1-5	16674257	16287442	226006	1.39%
slb1-5-2A	slb1-5	17775561	17343817	70128	0.40%
slb1-5-3A	slb1-5	17354884	16947949	60198	0.36%

Supplementary Table 5 The list of oxidative stress and transmembrane transporter related genes in darkgreen module identified by WGCNA analysis. The genes are listed with information including function category, protein function and expression profile (FPKM average by sample group).

Module	Function category	Gene name	Function	Transcript ID simplifier	Expression level									
					WT-S1	WT-S2	WT-S3	WT-S4	WT-S5	slb1-S1	slb1-S2	slb1-S3	slb1-S4	slb1-S5
Darkgreen module	Transmembrane transporter activity	PLT5	Polyol/monosaccharide transporter	c009732_t	4.5	6.2	3.0	6.1	3.2	10.7	16.4	2.5	5.1	3.1
				c140878_t	21.7	10.8	8.7	18.6	9.8	22.2	39.3	13.2	17.5	16.7
		PHT1;4	High-affinity transporter for external inorganic phosphate	c063555_t	3.2	7.1	13.6	31.2	14.9	38.6	121.5	49.1	42.2	24.9
				c115752_t	0.0	0.0	0.0	0.1	0.1	122.0	346.1	135.5	74.8	74.6
				c120920_t	0.0	0.0	0.0	0.0	0.0	59.5	211.1	55.5	31.8	18.7
				c135971_t	0.2	0.9	0.4	0.0	0.4	82.3	212.9	47.6	90.7	49.0
	Response to oxidative stress; MAPK signaling pathway	CAT2	Catalase	c058745_t	21.5	14.6	15.6	26.4	16.2	17.7	130.1	36.1	32.2	19.3
				c065634_t	43.1	0.0	17.2	38.0	19.0	24.9	91.0	28.0	29.4	15.7
				c079113_t	282.4	241.7	97.6	167.8	98.5	273.0	755.5	140.4	143.4	123.7
				c120894_t	4.6	8.1	1.6	2.9	0.4	3.8	29.1	11.2	2.4	2.4
				c129462_t	228.3	24.8	44.0	118.8	63.5	260.2	862.2	174.3	184.1	145.1
				c139770_t	192.0	117.1	56.7	114.8	55.9	178.5	409.9	71.2	96.6	57.5

Supplementary Table 6 Primer sequences for quantitative real-time PCR (qRT-PCR).

qRT-PCR analyses were performed with AceQ® qPCR SYBR® Green Master Mix (without ROX) (Vazyme, China). and run on an LightCycler 480 Ⅱ system (Roche, Switzerland) with the following programs: activation for 5 min at 95◦C, followed by 50 cycles of 10 s at 95◦C, 15 s at 60◦ C, and 15 s at 72◦ C.

Number	Unigene	Gene name	F	R
1	c129704_t	ABCB1	GAACTCAGCTCTCATGCTCGTT	TGCGGCAACTGTTCGAACATTG
2	c102483_t	ABCB19	GTGGCTTTCATTGTCGAATGGC	ATCTTGTCCTGGGCATTGAAGG
3	c063048_t	SUS4	AGCTCAAATGAATAGGGTGCGC	TAGGGATCAATGTGGAAGCCTG
4	c012890_t	SEP3	GAAGGAATGCAGGCAAATCCAC	CAGGTGCAGTGCTCATCGTTAT
5	c061666_t	ASK1	TTGGGTACCCCAACAAGAGAG	TCCAGAGCAGTACAACGTAGGT
6	c084733_t	FT	CAGGTGCAAGTTTCGGACAAGAG	CAACTGGCAATCCCAGGTTGTAG
7	c026545_t	FT	GCGATCCAACGCTAAGGGAGTAT	TGTCCGAAGATGGGCCTTACAGT
8	Reference gene	LcACT97	TTCCCGTTCAGCAGTGGTCG	TGGTCGCACAACTGGTATCG
9	Reference gene	LcUBQ10	ATTCCAGAGGACCAGTTCCTG	AGCAAGTGAGAGATTGTCCTTG

Supplementary Table 7 Sequences of primers for detection of alternative splicing of *FT*. RT-PCR amplification for the two splice variants of *FT* were performed using 2 × Rapid Taq Master Mix (Vazyme, China). PCR reaction conditions included denaturation at 95 °C for 3 min; followed by 30 cycles (95 °C for 15 s, 59 °C for 15 s, and 72 °C for 10 s; and a final extension at 72 °C for 5 min).

Number	Unigene	Primer	Primer Sequence
1	c084733_t	F1	TTCTACACACTGGTCATGGTGG
		R	CCTTCTTCCACCAGAACCTGTCT
2	c026545_t	F2	AGAGAACCTTTGGTTGTTGGCC
		R	CCTTCTTCCACCAGAACCTGTCT
